# Faster to react, slower to move: differential effects of multisensory facilitation on movement initiation and execution across the adult lifespan

**DOI:** 10.3389/fpsyg.2026.1811531

**Published:** 2026-09-09

**Authors:** Ayla Barutchu, Charles Spence

**Affiliations:** 1 Department of Experimental Psychology, University of Oxford, Oxford, United Kingdom; 2 Institute of Mental and Physical Health and Clinical Translation (IMPACT), Deakin University, Geelong, VIC, Australia

**Keywords:** action, ageing, attention, audiovisual, audition, motor planning and execution, multisensory integration, vision

## Abstract

**Introduction:**

Spanning multisensory integration, ageing, attention, and motor control, this study is, to our knowledge, the first to examine how audiovisual integration differentially influences the initiation and execution of goal-directed motor actions across the adult lifespan.

**Methods:**

Young adults (<30 years) and older adults (between 50 and 69 years and ≥70 years) completed a simple detection task and a visual discrimination task on a portable touchscreen device. In the detection task, participants tapped to indicate the perceived location of auditory, visual, or audiovisual stimuli. In the discrimination task, they responded to a visual target presented either with or without distractors, accompanied by auditory stimulation that was either spatially aligned or misaligned with the target.

**Results:**

Across both tasks, multisensory stimulation significantly reduced reaction times in younger and older adults, whereas movement speed slowed modestly, most reliably in the detection task and under audiovisual distraction during visual discrimination. Distractors disproportionately impaired performance in older adults, but spatially aligned auditory information partly alleviated this effect, especially in the oldest group. Importantly, these modest movement-speed costs did not outweigh the overall facilitation of goal-directed actions.

**Discussion:**

The results suggest that multisensory signals enhance rapid motor planning and initiation, followed by a later calibration phase that improves precision during movement execution. They also indicate that the spatial window of multisensory integration may narrow in the presence of distractors, particularly in older adults. Taken together, these findings demonstrate that multisensory facilitation remains functionally advantageous even under distracting conditions, albeit at a relatively small task-dependent cost to movement speed.

## Introduction

Multisensory integration refers to the process by which information from different sensory modalities, such as vision and audition, are combined to form a unitary representation. By enhancing the detectability, localisation, discrimination, learning, memory, and motor performance, multisensory processing benefits behaviour across the lifespan (e.g., Barutchu et al., 2009; Barutchu et al., 2020; Brunetti et al., 2017; Laurienti et al., 2006; Lovelace et al., 2003; Seitz et al., 2006; Stein et al., 1996). Despite these well-established advantages, most multisensory research relies on reaction times (RTs) recorded from simple button- or key-press tasks (e.g., Hillyard et al., 2016; Miller, 1982; Spence and Driver, 2004). Although these measures capture response initiation, they provide little information about the execution of the subsequent movement. This is an important limitation, because many daily actions require continuous, goal-directed motor control. For example, answering a phone, withdrawing from a barking dog, or shifting the foot between the accelerator and brake all require rapid coordinated movement after the response has been initiated. Whether audiovisual stimuli also influence movement speed, for example, remains poorly understood, particularly in touchscreen-based tasks where actions involve a lift from a resting position and a spatially directed tap, akin to many everyday touchscreen interactions.

Multisensory effects on motor response are highly task-dependent. In simple detection paradigms, where participants respond to both auditory and visual targets with the same motor action (e.g., using the same button), audiovisual stimuli produce faster and less variable RTs, a phenomenon also known as the redundant signals effect (Miller, 1982). Race models assume that the faster of two independently processed sensory signals initiates the response, such that multisensory RTs are faster than unisensory RTs purely because either sensory modality can trigger the response by chance (Giray and Ulrich, 1993; Miller, 1982; Raab, 1962). When multisensory RTs exceed this prediction (i.e., violate the race model), the findings are taken as evidence that the sensory signals interact rather than being processed independently. Importantly, the magnitude of multisensory facilitation is strongly influenced by task demands. For example, facilitation is substantially reduced when one sensory modality is task irrelevant and participants are instructed to respond only to the task-relevant modality (Barutchu and Spence, 2020, 2021; Giray and Ulrich, 1993; Miller, 1982). Meanwhile, when separate motor responses are required for auditory and visual stimuli, the Colavita effect sometimes emerges, whereby participants may fail to detect or report the auditory component of multisensory stimuli, underscoring the sensitivity of multisensory processing to task and motor demands (Sinnett et al., 2008). The motor network itself appears to contribute to the calibration of multisensory processes (Bischoff et al., 2014; Huang et al., 2015; Murakami et al., 2018), indicating that interactions between sensory and motor systems are central to these effects. Similarly, in a simulated driving task, audiovisual stimuli produced comparable multisensory facilitation during goal-directed actions such as lifting the foot from the accelerator to the brake (Levy et al., 2006), suggesting that lifting and button-pressing may rely on comparable underlying mechanisms. More recently, studies have demonstrated that auditory cues can also improve the temporal and spatial characteristics of goal-directed movements, including movement accuracy, timing, and online movement control (Kreyenmeier et al., 2024; Malouka et al., 2023; Peters and Glazebrook, 2020). Studies of target-directed reaching, primarily using visual–haptic integration, further suggest that sensory information is optimally combined to minimise motor noise, possibly through a two-stage process involving an initial impulse phase followed by a later calibration phase that controls limb-to-target movement (e.g., Camponogara, 2023; Ernst and Banks, 2002; Todorov and Jordan, 2002). However, relatively little is known about how audiovisual (i.e., multisensory) targets influence both movement initiation and movement execution during goal-directed actions, particularly across the adult lifespan and under differing attentional demands.

The role of spatial coincidence in multisensory integration also varies considerably across tasks. While facilitatory effects reliably depend on the temporal synchrony of signals at both behavioural and neural levels, the role of spatial coincidence is more task dependent (e.g., Bruns and Roder, 2023; Spence, 2013, 2025; Stein and Meredith, 1993; Zuanazzi and Noppeney, 2020). Animal studies provide strong support for the spatial rule, whereby neuronal integration is enhanced when stimuli originate from the same spatial location (Stein and Meredith, 1993), whereas human evidence suggests that spatial coincidence is most important when tasks require overt or covert orienting or spatial discrimination, but less so for non-spatial tasks (Doyle and Snowden, 2001; Spence, 2013, 2025; Spence and Driver, 1996, 1997). Spatial modulation also becomes more prominent when targets are likely to appear in an expected location (Spence and Driver, 2004; Zuanazzi and Noppeney, 2019, 2020), when targets are masked or spatially uncertain (Bolognini et al., 2010), and when sensory reliability differs between modalities, as demonstrated by the ventriloquist effect (Alais and Burr, 2004). Similarly, multisensory spatiotemporal coincidence and the locus of gaze matter more in the presence of distractors as seen in the cocktail party effect (e.g., Ahmed et al., 2023a; Ahmed et al., 2023b; Jia et al., 2023), suggesting that the spatial locus of attention also plays an important role in multisensory integration. Importantly, both animal and human studies also point to a degree of spatiotemporal tolerance that likely facilitates the integration of signals originating from spatially separated sources (e.g., Diederich and Colonius, 2015; Stein and Meredith, 1993; Stein and Stanford, 2008; Stevenson et al., 2012). The influence of spatially orientated goal-directed motor requirements on multisensory integration, particularly their effects on movement dynamics beyond initial RTs, remains largely unexplored.

One attribute of attention, namely, the ability to focus on relevant information while suppressing distractors, has been shown to modulate multisensory integration (e.g., Barutchu et al., 2019; Buchan and Munhall, 2011; Downing et al., 2014; Driver and Spence, 1998; Fairhall and Macaluso, 2009; Talsma et al., 2007; Talsma et al., 2010). Selective attention prioritises processing in the attended modality while slowing down processing in the unattended modalities (Driver and Spence, 1998; Spence and Driver, 1996, 1997; Spence et al., 2001). For example, when a spatially non-informative auditory signal is temporally, but not spatially, aligned with a visual target among distractors, visual search times are reduced (Klapetek et al., 2012; Van der Burg et al., 2008). In this case, selective spatial attention facilitates the binding of temporally synchronous auditory and visual signals into a salient event, allowing targets to “pop out” phenomenologically. Conversely, directing attention to a single sensory modality or away from the multisensory event reduces multisensory integration (Talsma et al., 2007; Talsma et al., 2010), and both unisensory and multisensory task-irrelevant distractors slow responses in visual discrimination tasks (Barutchu et al., 2013; Barutchu and Spence, 2021; Downing et al., 2014; Molholm et al., 2004). Irrelevant auditory stimuli typically interfere less with visual target detection than visual distractors interfere with auditory target detection (Downing et al., 2014; Hillyard et al., 2016; Molholm et al., 2004), consistent with the omnidirectional nature of sound, which can orient attention towards events outside the relatively constrained visual field. Conversely, during object discrimination, vision tends to dominate over audition, potentially increasing susceptibility to coincident task-irrelevant auditory signals and visual distractors. Together, these findings highlight the complex, bidirectional relationship between attention and multisensory integration (Spence, 2018). On the one hand, multisensory integration depends on attention, as task-relevant multisensory signals are preferentially integrated. On the other, multisensory signals can capture, redirect, and facilitate attention, thereby enhancing performance in everyday environments that are often cluttered with competing sensory information.

Multisensory benefits are not confined to young adults but are sometimes amplified in older adults, particularly for RTs (Couth et al., 2018; de Dieuleveult et al., 2017; Laurienti et al., 2006; Mahoney et al., 2012; Mozolic et al., 2012; Peiffer et al., 2007). For example, Laurienti et al. (2006) found that multisensory targets facilitated discrimination responses more in older adults than in younger adults, and that these gains were independent of general cognitive slowing (Cerella, 1985). Similarly, Peiffer et al. (2007) found that when unisensory RTs were matched across groups, older adults still demonstrated greater multisensory benefits, indicating that these gains cannot be explained by global slowing alone. However, more recent evidence suggests that enhanced multisensory facilitation is not consistently observed across all tasks or experimental paradigms (Atkin et al., 2024; Jones and Noppeney, 2021; Roudaia et al., 2018). One potential explanation for these inconsistencies is the considerable heterogeneity within older adult populations. Most previous studies have compared young adults with a single broad older adult group (typically spanning 60–80 years or more), potentially obscuring stage-based changes across later adulthood (de Dieuleveult et al., 2017; Jones and Noppeney, 2021).

Age-related enhancements in multisensory processing may be explained by the principle of inverse effectiveness, which predicts stronger multisensory facilitation when unimodal signals are weak or degraded (Lovelace et al., 2003; Stein et al., 1996; Stein and Meredith, 1993), although these effects may also be influenced by changes in response bias and the reliability of sensory inputs (Odgaard et al., 2003). With sensorimotor and cognitive function progressively declining with age (Humes et al., 2013; Park and Reuter-Lorenz, 2009), older adults may rely more heavily on combined sensory input to support stable perception (Cienkowski and Carney, 2002; Schulte et al., 2020). Consistent with this interpretation, multisensory effects, including perceptual phenomena such as the McGurk effect, are often more prominent in individuals with visual and hearing impairments (Frassinetti et al., 2005; Schulte et al., 2020). Age-related differences in selective attention and distractor processing have also been reported, although these effects are task dependent and not consistently observed across paradigms (Alain and Woods, 1999; Guerreiro et al., 2010; Park and Reuter-Lorenz, 2009; Rey-Mermet and Gade, 2018). Reduced selective control may therefore allow unattended sensory inputs to exert a greater influence on perception, particularly under conditions of increased attentional demand. Indeed, attentional load can modulate distractor effects: incongruent distractors disrupt visual search performance under low load when attentional resources spill over to irrelevant inputs (Brand-D'Abrescia and Lavie, 2008; Lavie, 1995), whereas these effects diminish under high load as spatial attention narrows, although crossmodal distractors often continue to interfere, suggesting that auditory and visual inputs engage partially distinct attentional resources (Duncan, 1980; Matusz et al., 2015; Tellinghuisen and Nowak, 2003). Thus, reduced selective control in ageing may paradoxically amplify multisensory integration by enabling a greater influence of unattended sensory signals. This apparent advantage suggests that multisensory stimuli may be particularly effective for enhancing attention, target detection, and discrimination in older adults by capitalising on temporal synchrony and distributed attentional resources. However, it remains unclear whether these benefits extend beyond faster response initiation to also improve movement execution and movement dynamics.

From both theoretical and practical perspectives, it is important to determine whether multisensory benefits and distractor costs extend beyond RTs to encompass the speed and precision of goal-directed movements, and how these effects generalise across the lifespan. We therefore examined multisensory facilitation in young adults (< 30 years) and two stages of later adulthood (50–69 years and ≥70 years) using a touchscreen platform capable of recording lift RTs, tap locations, and tap latencies, enabling estimates of movement dynamics, including speed and tap precision (relative to target location). Given the recognised heterogeneity of cognitive ageing, separating older adults into two age bands allowed us to evaluate potential stage-based changes in multisensory processing across later adulthood (Becker, 2010; National Academies of Sciences, 2015; Park and Reuter-Lorenz, 2009; Rodrigues et al., 2025; Salthouse, 2009). We expected multisensory facilitation to be optimal when both auditory and visual signals were task-relevant, and smaller, though still reliably detectable, when auditory stimuli were task-irrelevant or when visual distractors were present. Accordingly, participants completed two tasks: (i) a simple audiovisual detection task designed to replicate robust multisensory benefits, and (ii) a visual discrimination task with lateralized, irrelevant auditory stimuli and visual distractors, in which smaller multisensory gains were anticipated due to attentional competition.

Audiovisual stimuli were predicted to facilitate both movement initiation (lift RT) and movement completion (tap RT) relative to unisensory stimuli, with larger multisensory benefits in the detection than the discrimination task and in older than younger adults. Whether these benefits changed progressively across later adulthood and extended beyond movement initiation to influence movement speed was also examined. For the discrimination task, visual distractors were predicted to slow responses, whereas audiovisual spatial congruence was expected to partially offset these costs.

## Methods

### Participants

A total of 57 participants were recruited from the Oxford Cognitive Neuropsychology Centre, and subdivided into three age groups: <30yo group (*N* = 17, 6 males, 17 right-handed, *M* age = 24.29 years, *SD* = 2.14, range = 21–27 years), 50–69yo group (*N* = 18, 11 males, 14 right-handed, *M* age = 63.22 years, *SD* = 5.69, range = 49–69 years) and ≥70yo group (*N* = 21, 8 males, 20 right-handed, *M* age = 74.67 years, SD = 3.64, range = 70–83 years) (Becker, 2010). One participant aged 49 years was retained in the 50–69yo group as their age borders the ‘young old’ stage of development and because their age fell within two standard deviations of the group mean. The G*Power program was used to run power analyses to estimate the required sample size (Faul et al., 2007). Based on previous studies demonstrating robust multisensory facilitation effects (Barutchu and Spence, 2021; Miller, 1982), a large effect size (*f* > 0.50) was anticipated. Assuming *α* = 0.05 and power = 0.80, the three-group mixed repeated measures ANOVA with 12 repeated conditions indicated a minimum sample size of 18 participants (6 per group). However, because no prior studies had examined the effects of age group on movement speed in this paradigm, it was not possible to reliably estimate the expected effect size for this outcome. We therefore adopted a more conservative recruitment target of at least 16 participants per group to ensure adequate power to detect age-related differences across the primary behavioural measures. To better reflect the final design, a *post hoc* sensitivity analysis was conducted using G*Power program, indicating that the study was adequately powered to detect small-to-large effect size (approximately *f* = 0.14 or larger).

All the participants self-reported normal or corrected to normal vision and hearing, reported no neurological or psychiatric disorders, and all gave their informed consent to take part in the experiments. All of the procedures were approved by the Research Ethics Committee at the University of Oxford.

### Stimuli and procedure

**Figure 1:**
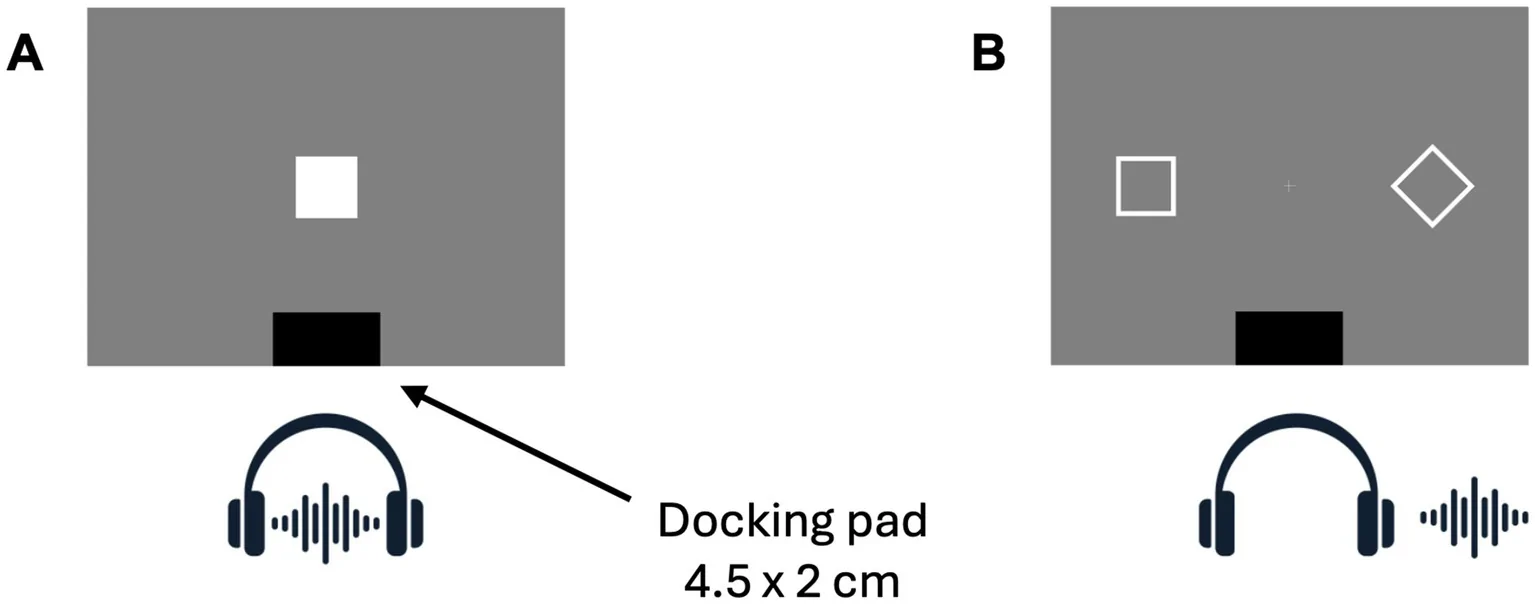
Illustration of the detection and discrimination tasks. The active display area measured 148 mm × 197 mm. Throughout each trial, a 1 cm × 1 cm fixation cross appeared at the centre of the screen, and the docking pad, where participants rested their finger between trials, measured 4.2 cm× 2 cm. All target stimuli measured 2.5 cm^2^ and differed only in their orientation. **(A)** Representation of an audiovisual stimulus in the simple detection task. **(B)** Representation of the visual discrimination task, in which participants were asked to tap on a visual target shape (diamond or square, counterbalanced across participants) as quickly as possible. Stimulus conditions included targets were presented on the left or on the right, with or without a distractor and auditory sound. This example shows the Aic-Vd condition, where the visual target was the diamond, presented with a spatially congruent auditory stimulus and a visual distractor on the opposite side.

The task design and parameters were informed by iterative piloting across the adult lifespan to maximise accuracy, and in turn, accessibility while maintaining sensitivity to multisensory and attentional processes. The participants completed two tasks in sequence: a multisensory detection task followed by a visual discrimination task; both administered on a portable touchscreen device (an iPad with an active display area: 148 mm × 197 mm). Testing took place in a quiet, sound-attenuated, dimly-lit room. The participants were seated in a comfortable chair with the touchscreen placed in landscape orientation on a waist-height table, aligned with their body’s midline. A white fixation cross (1.0 cm × 1.0 cm) was continuously displayed at the centre of a grey screen, with a black rectangle (4.5 cm × 2.0 cm), referred to as the ‘docking pad,’ positioned centrally along the horizontal midline (see Figure 1A).

Across both tasks, auditory stimuli consisted of a 1 kHz pure tone (70 dB, 5 ms onset/offset ramp) presented through closed headphones. The synchrony between the auditory and visual stimuli in the audiovisual trials was verified using an oscilloscope, which confirmed less than 2 ms onset jitter.

Participants responded with their dominant hand. At the beginning of each trial, the participants rested their index finger on the docking pad. They were instructed to lift their finger upon target presentation, tap the centre of the target, and return their finger to the docking pad until the next trial. If the finger was not docked at trial onset or was lifted prematurely, the response was coded as a docking error. For each trial, RTs and the spatial coordinates of finger lift and tap responses were recorded. The gesture recognition of the touchscreen was set at 120 Hz. Stimuli were presented for 200 ms with an interstimulus interval of 3–4 s. Before each task, participants completed a minimum of 15 practice trials and continued until they achieved at least 80% accuracy. All participants reached criterion within 50 trials per task. During testing, the experimenter was seated behind (and to the side) of participants outside their field of vision.

### Audiovisual detection task

In the detection task, participants were presented with auditory (AT), visual (VT), and audiovisual (AVT) targets. The visual stimulus was a 2.5 cm^2^ square presented at fixation (Figure 1B). Initially, a solid white square was used; however, some participants found it too bright. To address this issue, the stimulus was changed to a royal blue square naturally of lower contrast, but still easily perceptible, for 11 of the participants. Preliminary analyses were conducted to compare participants tested before and after the colour change. Independent-samples *t*-tests, 95% confidence intervals (CI), and inspection of the directionality of multisensory gains and distractor costs indicated no meaningful differences between groups; therefore, the data were combined for all subsequent analyses.

Auditory stimuli were delivered binaurally at equal intensity to create the percept of a centrally-located sound. For AVT trials, auditory and visual stimuli were presented simultaneously. A total of 90 trials was administered, divided into six blocks of 15 trials. The three stimulus types (AT, VT, and AVT) occurred randomly and with equal probability.

### Visual discrimination task

In the visual discrimination task, participants discriminated between a square and a diamond (2.5 cm^2^, 2 mm line thickness), which were physically identical except for their orientation (see Figure 1B). At task onset, one shape was designated as the target and the other as the distractor, counterbalanced across participants. Stimuli were presented 5.8 cm to the left or right of fixation, either alone or with a 1 kHz tone presented to the left or right ear. The auditory stimulus could be spatially congruent or incongruent with respect to the target side. The participants were instructed to respond to visual targets only, while ignoring any auditory stimuli and the visual distractors.

The task included 21 stimulus conditions. Twelve of these corresponded to target conditions, which included visual targets presented alone (VT), visual targets presented with a visual distractor (Vd), visual targets accompanied by a spatially congruent auditory stimulus (Ac), visual targets accompanied by a spatially incongruent auditory stimulus (Aic), visual targets paired with a congruent auditory stimulus and a distractor on the opposite side (Ac-Vd), and visual targets paired with an incongruent auditory stimulus presented on the same side as the distractor (Aic-Vd). The remaining nine conditions were invalid distractor trials, defined by the combination of auditory presentations (none, left ear, right ear) and distractor position (left, right, or bilateral).

Stimuli were presented randomly across six blocks of 75 trials. Target trials accounted for 80% of stimuli, presented with equal probability (30 per target condition, distributed evenly across blocks). The remaining 20 percent of stimuli were invalid distractors, which were randomly intermixed throughout the task. On rare occasions, the low probability of invalid stimuli meant that some conditions were not represented. To address this, the task was amended for 11 participants so that invalid stimuli were presented at approximately equal probabilities across conditions. Note that the number of target and invalid stimuli remained the same across all participants. Comparisons of participants tested before and after this minor procedural amendment revealed no meaningful differences in any primary outcome measure; therefore, the data were combined for all subsequent analyses.

### General data processing and analysis

For both the detection and visual discrimination tasks, lift RTs and tap times (both measured in ms from stimulus onset) were recorded, together with the x-y coordinates of lift and tap locations, which were measured in pixels and converted to millimetres (mm). Only valid trials, defined as those in which participants were correctly docked at trial onset and able to maintain finger position on the docking pad between trials, were included in the analyses. Trials with multiple taps on the docking pad were classified as docking errors, which were recorded separately for each trial.

For each valid trial, lift errors were defined as either missed responses, false alarms to invalid stimuli in the visual discrimination task, or trials with lift RTs < 100 ms or exceeding 3 standard deviations (*SD*) from the participant’s mean. Tap errors were defined as trials in which a correct lift was made but (1) the target was missed, (2) the tap latency exceeded 3 *SD* from the participant’s mean, or (3) the tap location deviated by more than 3 *SD* from the participant’s mean distance from the target. For the visual discrimination task, it was further verified that taps were consistently directed to the correct target side relative to the central fixation cross. Each trial was classified as either correct or incorrect, such that no trial was counted more than once in the overall error measure. Both the detection and visual discrimination tasks were designed to be readily performed across the adult lifespan, and error rates were therefore very low. As each individual error type occurred infrequently, all incorrect trials were combined to calculate a single percentage error measure within each task and age group for analysis.

An inclusion criterion of at least 50% accuracy per target condition was applied, ensuring a minimum of 15 valid samples per condition to provide reliable lift RT and tap measures. The distance travelled between lift and tap, together with the movement time, was used to calculate movement speed (i.e., the absolute magnitude of velocity expressed as mm/ms), thereby controlling for variability in the distances covered due to the size of the target and the docking area. The distance between the tap location and the central point of the target object in the discrimination task, and between the tap location and the fixation cross in the detection task, were also calculated. For each participant, mean values were computed for lift RTs, tap times, movement speed, and the tap distance from the target location. Because the tap time results closely mirrored those for lift RTs, only the lift RT and movement speed analyses are presented here. The corresponding tap time analyses are reported in the Supplementary Informations B, D.

Participants consistently tapped within target boundaries, indicating high motor accuracy across all conditions. Tap distance from the centre of the target did not differ significantly across age groups or audiovisual conditions. The only significant effect was observed in the visual discrimination task, where distractors slightly reduced the spatial accuracy of the taps; In the absence of distractors, taps were closer to the target by less than 2 mm on average (Supplementary Information A). Given the small effect size and limited functional relevance, tap distance from the target was not analysed further.

For each participant and task, cumulative distribution functions (CDFs) were computed for lift RTs and tap times on correct target trials. CDFs represent the probability of observing a RT at or below a given time point. Cumulative probabilities were calculated from 0.01 to 0.95 in increments of 0.1, with a maximum probability of 1 indicating that all motor responses within a condition were faster than the specified time point.

Mean lift RTs and movement speed were analysed using mixed-design Analyses of Variance (ANOVAs). Where appropriate, significant effects were followed by analyses of simple main effects and pairwise comparisons. Mauchly’s test was used to assess sphericity, and Greenhouse–Geisser corrections were applied where violations were detected. All reported results have been corrected for multiple comparisons using Šidák corrections.

## Results

### Accuracy for the audiovisual detection task

Data from two participants in the <30yo group were not recorded, leaving 15 participants in this group for the detection-task analyses.

To determine whether multisensory facilitation reflected a speed-accuracy trade-off, response accuracy was analysed. Accuracy on the simple detection task was uniformly high. The median percentage error rate for most stimulus conditions across all groups was zero, violating the assumption of normality (see Table 1). Accordingly, non-parametric analyses were performed. Friedman tests revealed no significant effect of stimulus condition in the <30-year group, *χ^2^*(2) = 2.61, *p* = 0.27, or the 50–69-year group, *χ^2^*(2) = 5.02, *p* = 0.08. Although the omnibus Friedman test was significant in the >70-year group, *χ^2^*(2) = 9.57, *p* = 0.008, no pairwise comparisons remained significant following correction for multiple comparisons. Kruskal–Wallis tests revealed no significant differences in error rates between age groups for any stimulus condition: AT: H(2) = 5.24, *p* = 0.07; VT: H(2) = 3.10, *p* = 0.21; AVT: H(2) = 5.16, *p* = 0.08.

**Table 1:** Means (M), medians (Med) and standard deviations (SD) percentage (%) errors for overall error rates, between group independent samples Kruskal-Wallis tests comparing the three groups (<30yo, 50–69yo, and ≥70yo) and related samples Friedman’s tests (χ2) comparing errors percentage error rates within each group.

Stimuli	<30yo			50–69yo			≥70yo			Kruskal-Wallis
	*M*	*Med*	*SD*	*M*	*Med*	*SD*	*M*	*Med*	*SD*	
AT	1.44	0	1.83	3.11	1.72	4.58	4.99	3.70	7.47	H(2) = 5.24, *p* = 0.07
VT	1.21	0	1.77	0.84	0	1.64	2.28	0	2.94	H(2) = 3.10, *p* = 0.21
AVT	0.48	0	1.26	2.20	0	4.08	2.76	0	4.69	H(2) = 5.16, *p* = 0.08
	*χ*2 (2) = 2.61, *p =* 0.27			*χ*2 (2) = 5.02, *p =* 0.08			*χ*2 (2) = 9.57, *p =* 0.008			

### Motor responses for the audiovisual detection task

**Figure 2:**
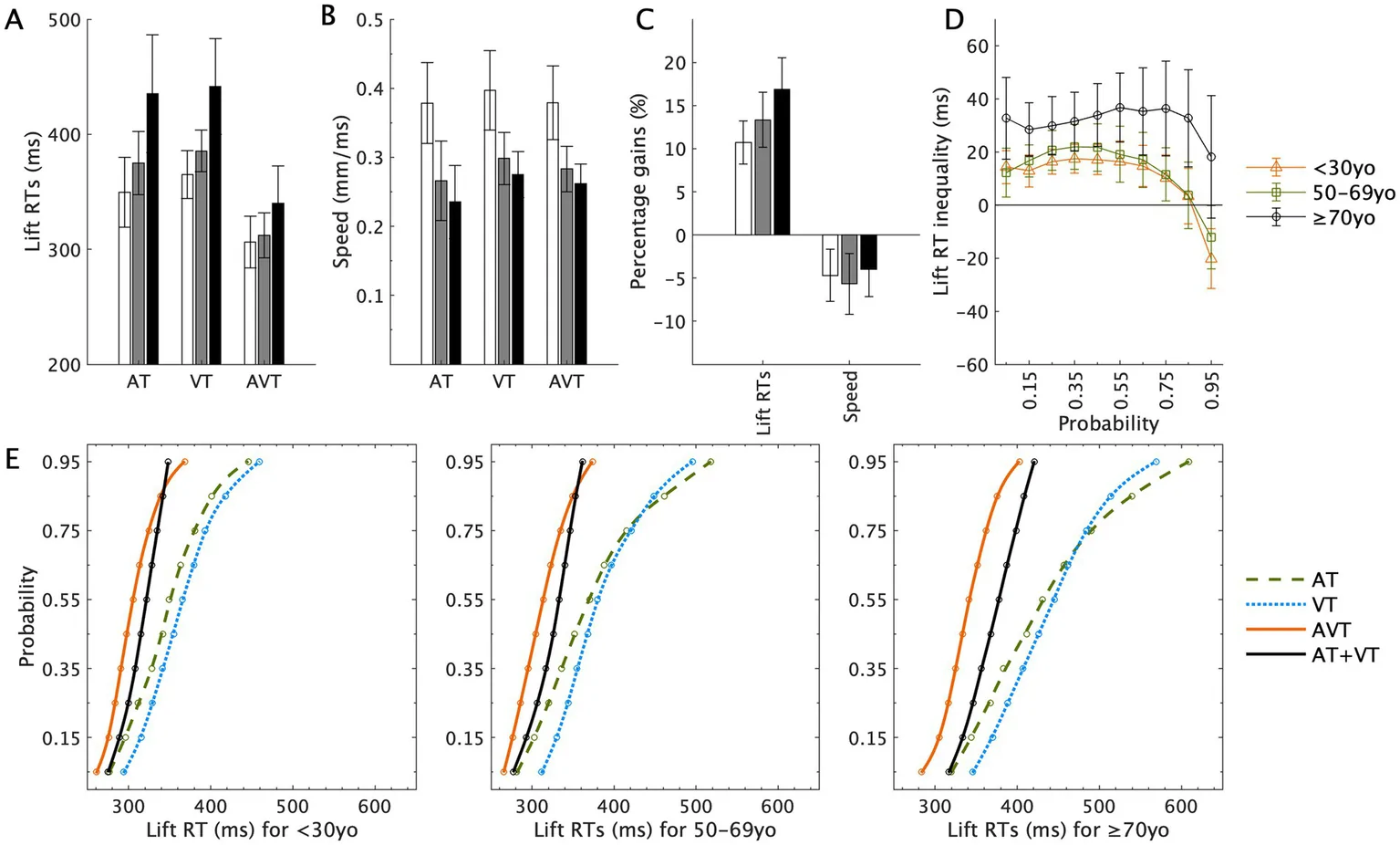
Motor responses on the detection task. **(A)** Mean lift RTs (ms) and **(B)** speed (mm/ms) for auditory (AT), visual (VT), and audiovisual (AVT) stimuli for each group: <30yo (white), 50–69yo (grey), and ≥70yo (black). **(C)** Mean multisensory gains relative to the faster of the unisensory conditions as measured in milliseconds (ms) for lift RTs and speed percentage gains (%). **(D)** Inequality in ms between AVT CDF and AT+VT CDF with positive values representing race-model violations. **(E)** Cumulative density functions (CDFs) for lift RTs for AT, VT, AVT, and AT+VT CDFs for each age group. Error bars represent 95% CI.

To determine whether multisensory facilitation influenced both movement initiation and execution, lift RTs and movement speed were analysed separately. The fastest lift RTs were observed for multisensory stimuli; however, such multisensory enhancements were not observed for speed (see Figure 2). Two 3 (age groups) x 3 (stimuli type: AT, VT, and AVT) mixed design ANOVAs were conducted to analyse lift RTs and movement speed, followed, where appropriate, by analyses of main effects and simple pairwise comparisons.

In general, although lift RTs increased with age, audiovisual facilitation of lift RTs was evident across all age groups (Figure 2A). There was a significant main effect of age group, *F*(1, 51) = 5.75, *p* = 0.006, partial *η^2^* = 0.18, with the <30yo group responding faster overall than the ≥70yo groups (*p* = 0.008). There was also a significant main effect of stimulus type, *F*(1.67, 85.51) = 102.54, *p* < 0.001, partial *η^2^* = 0.67, reflecting faster lift RTs to multisensory (AVT) than unisensory stimuli (*p* < 0.001 for both). Lift RTs to the two unisensory stimuli did not differ significantly (all *p* > 0.05). Importantly, the Age Group × Stimulus Type interaction was also significant, *F*(3.35, 85.51) = 3.94, *p* = 0.009, partial *η^2^* = 0.13. Follow-up simple effects analyses showed that the ≥70yo group responded significantly more slowly than the <30yo group in the AT (*p* = 0.008) and VT (*p* = 0.002) conditions. In addition, the 50–69yo group was significantly slower than the <30yo group in the VT condition (*p* = 0.02). In contrast, lift RTs to the multisensory (AVT) stimuli did not differ significantly between the three age groups (all *p* ≥ 0.05).

The pattern of results differed for movement speed (Figure 2B). There was a significant main effect of stimulus type, *F*(1.58, 80.63) = 23.68, *p* < 0.001, partial *η^2^* = 0.32. The fastest movement speeds were recorded for the unisensory VT stimulus (for both VT vs. AT and AVT, *p* < 0.001), followed by the AVT stimulus (for VT vs. AVT, *p* < 0.001), with the slowest movement speeds observed for the AT stimulus (*p* = 0.004 for AT vs. AVT). There was also a significant main effect of age group, *F*(1, 51) = 13.62, *p* < 0.001, partial *η^2^* = 0.35, with the <30yo group moving significantly faster than both the 50–69yo and ≥70yo groups (*p* < 0.001 for both comparisons), whereas the two older groups did not differ significantly from each other (*p* = 0.66). The Age Group × Stimulus Type interaction was not significant, *F*(3.16, 80.63) = 1.65, *p* = 0.17, partial *η^2^* = 0.06. Despite movement speed being slightly faster for the VT than the AVT stimulus, robust multisensory facilitation was still observed for tap RTs (i.e., at movement completion before the participant’s finger returned to the docking pad; see Supplementary Information B).

#### Multisensory facilitation in the detection task

To account for baseline differences in response speed and permit direct comparisons across behavioural measures and age groups, percentage gains (or costs) were calculated. For each participant, percentage gains (or costs) in lift and tap times (ms) and movement speed (mm/ms) for multisensory stimuli were calculated by first identifying, for each participant, the faster mean response of either the AT or VT condition and comparing it with the corresponding multisensory (AVT) response. These differences were then expressed relative to the faster unisensory condition. The percentage gain was computed as:


$$\text{Percentage Gain}=\frac{\left(\text{Faster Unisensory}-\mathrm{AVT}\right)}{\text{Faster Unisensory}}\times 100$$

Note that the formula was adjusted so that positive values consistently represented multisensory gains. For lift and tap RTs, positive values indicated shorter RTs for the multisensory condition relative to the faster of the two unisensory conditions. For movement speed, where higher values indicate better performance, the direction of the formula was reversed so that positive values likewise indicated multisensory gains (gains in tap times are presented in Supplementary Information B). As can be seen in Figures 2C and D, significant multisensory gains averaging over 10% were observed for lift RTs, whereas movement speed was significantly reduced under multisensory conditions by approximately 5% across all age groups. To assess group differences across these measures, we conducted a 2 (measure: lift RT and movement speed) × 3 (age group: <30yo, 50–69yo, and ≥70yo) mixed-design ANOVA. There was a significant main effect of measure, *F*(1, 51) = 224.60, *p* < 0.001, partial *η^2^* = 0.82, with percentage multisensory gains being significantly greater for lift RTs, whereas movement speed showed multisensory costs. Neither the main effect of age group, *F*(2, 51) = 2.56, *p* = 0.09, partial *η^2^* = 0.09, nor the Measure × Age Group interaction, *F*(2, 51) = 1.66, *p* = 0.20, partial *η^2^* = 0.06, was significant. One-sample *t*-tests against zero confirmed significant lift RT gains in all three age groups: <30yo, *t*(14) = 9.25, *p* < 0.001; 50–69yo, *t*(17) = 8.78, *p* < 0.001; and ≥70yo, *t*(20) = 10.15, *p* < 0.001. Conversely, movement speed was significantly reduced in all age groups: <30yo, *t*(14) = −3.34, *p* = 0.005; 50–69yo, *t*(17) = −3.39, *p* = 0.003; and ≥70yo, *t*(20) = −2.61, *p* = 0.017.

Nevertheless, the observed multisensory gains may not necessarily reflect genuine multisensory integration but instead could arise from probabilistic facilitation produced by two competing sensory signals, as described by race models (i.e., statistical facilitation). Race models assume that the fastest motor responses under multisensory conditions (as reflected in the AVT CDF) cannot exceed the summed probabilities of the corresponding unisensory conditions (i.e., AT CDF + VT CDF). To test for violations of this assumption, the race model CDF (AT CDF + VT CDF) was subtracted from the AVT CDF, producing a difference curve (see Gondan, 2009; Miller, 1982, 1986, 2016; Ulrich et al., 2007 for a detailed outline of race models and test of inequality). Positive deviations indicate that AVT responses were faster than predicted by statistical facilitation, suggesting true multisensory integration, whereas negative deviations imply that AVT responses are consistent with statistical facilitation and thus explained by race models (see Figures 2B,D). Because race model violations are expected to occur primarily among the fastest responses, the race model was considered violated when positive deviations were observed for at least two consecutive cumulative probabilities below 0.5 (Barutchu et al., 2011). Race model violations were observed for all lift RTs below 0.5 probability for all three groups (see Figures 2D,E). A 3(age group: <30yo, 50–69yo, and ≥70yo) x 5(probability) mixed ANOVA showed significant main effect of group for lift RTs, *F*(2,47) = 5.07, *p* = 0.01, partial *η^2^* = 0.18. Race-model violations were significantly higher for the ≥70yo than the <30yo and 50–69yo group throughout the fast end of both the lift RT and tap distribution (*p* < 0.05 for all comparisons). The main effect for probability, *F*(1.76, 82.86) = 1.19, *p* = 0.32, partial *η^2^* = 0.03, and the interaction between probability and age groups were not significant, *F*(3.53, 82.86) = 0.30, *p* = 0.85, partial *η^2^* = 0.01.

This enhanced multisensory facilitation in older adults may be partly driven by sensory switching (Barutchu and Spence, 2020, 2021; Kreutzfeldt et al., 2015; Otto and Mamassian, 2012). The present study was designed to be easily completed by those with unstable selective attention; therefore, we do not have sufficient samples for a comprehensive, reliable sensory switch cost analyses. Nevertheless, a preliminary sensory switch cost analysis suggested that switch costs do indeed contribute to multisensory enhancement across the adult lifespan (see Supplementary Information C).

### Visual discrimination task

#### Accuracy for the visual discrimination task

One participant from the <30yo group, two from the 50–69yo group, and three from the ≥70yo group did not complete the discrimination task. An additional 50–69yo participant was excluded due to high error rates (>50%) in distractor conditions. Overall accuracy on the visual discrimination task was high across all age groups (Table 2). Error rates for target stimuli averaged below 6% across all stimulus conditions. Friedman tests revealed no significant differences in error rates across stimulus conditions within any age group (all *p* > 0.20), and Kruskal–Wallis tests showed no significant differences between age groups (all *p* ≥ 0.05).

**Table 2:** Means (M), medians (Med) and standard deviations (SD) overall percentage (%) errors for target and invalid stimuli (shaded in grey), between group independent samples Kruskal-Wallis tests comparing the three groups (<30yo, 50–69yo, and ≥70yo) for each stimulus, and related samples Friedman’s tests (χ^2^) comparing the 12 target and 9 invalid stimuli within each group.

VT	Vd	Aud	**<30yo**			**50–69yo**			**≥70yo**			Kruskal-Wallis
			*M*	*Med*	*SD*	*M*	*Med*	*SD*	*M*	*Med*	*SD*	
L	--	--	3.75	3.33	3.42	5.01	3.33	6.33	4.44	3.33	5.72	H(2) = 0.17, *p* = 0.92
L	--	Ac	3.13	3.33	2.57	5.63	3.33	7.27	4.27	5.00	3.77	H(2) = 0.67, *p* = 0.71
L	--	Aic	4.17	3.33	4.79	4.39	1.67	7.77	5.93	6.67	3.71	H(2) = 5.35, *p* = 0.07
L	R	--	2.50	1.67	3.10	8.75	5.00	12.29	5.56	3.39	5.11	H(2) = 6.00, *p* = 0.05
L	R	Ac	2.50	3.33	2.58	6.26	1.67	12.16	5.19	5.00	3.47	H(2) = 4.96, *p* = 0.08
L	R	Aic	2.29	0.00	3.15	8.13	3.33	16.95	4.46	3.39	3.43	H(2) = 4.22, *p* = 0.12
R	--	--	3.97	3.33	3.03	3.97	3.33	3.03	5.75	5.00	4.40	H(2) = 1.52, *p* = 0.47
R	--	Ac	4.18	3.33	3.12	4.17	3.33	4.47	5.75	3.39	4.09	H(2) = 2.16, *p* = 0.34
R	--	Aic	3.54	3.33	2.57	2.92	0.00	3.82	5.37	3.33	4.14	H(2) = 3.79, *p* = 0.15
R	L	--	4.79	3.33	4.38	3.13	3.33	4.47	3.72	3.33	3.42	H(2) = 2.37, *p* = 0.31
R	L	Ac	2.96	3.33	2.99	3.13	3.33	4.47	4.63	3.33	3.82	H(2) = 2.11, *p* = 0.35
R	L	Aic	4.79	3.33	2.97	3.97	3.33	4.90	5.37	5.00	3.05	H(2) = 3.12, *p* = 0.21
Friedman’s			*χ*2 (11) = 13.32, *p* = 0.27			*χ*2 (11) = 14.43, *p* = 0.21			*χ*2 (11) = 8.95, *p* = 0.63			
--	L	--	13.24	10.80	16.14	18.63	16.67	16.21	16.77	13.94	18.07	H(2) = 0.90, *p* = 0.64
--	L	L	15.84	10.10	19.90	24.92	17.42	28.60	28.39	22.50	24.63	H(2) = 3.51, *p* = 0.17
--	L	R	8.54	8.01	10.64	26.60	20.20	24.05	25.93	23.61	22.82	H(2) = 7.40, *p* = 0.02*
--	R	--	9.90	7.11	14.24	19.05	17.42	19.06	15.86	16.67	13.02	H(2) = 5.71, *p* = 0.06
--	R	L	8.42	4.17	11.12	26.74	11.81	32.30	25.74	22.50	21.02	H(2) = 9.46, *p* = 0.009*
--	R	R	12.22	9.09	11.56	21.58	13.33	23.79	26.38	20.00	18.59	H(2) = 4.53, *p* = 0.10
--	B	--	11.91	0.00	23.23	17.59	12.50	20.16	18.84	16.67	17.29	H(2) = 2.92, *p* = 0.23
--	B	L	17.03	10.00	23.92	14.89	9.55	18.53	31.68	26.67	26.30	H(2) = 3.37, *p* = 0.19
--	B	R	18.16	12.70	23.70	20.76	17.42	25.05	25.74	27.27	14.87	H(2) = 2.35, *p* = 0.31
Friedman’s			*χ*2 (8) = 8.31, *p* = 0.40			*χ*2 (8) = 11.19, *p* = 0.19			*χ*2 (8) = 14.88, *p* = 0.06			

VT, visual target; Vd, visual distractor; Aud, auditory signal; Ac, Auditory congruent; Aic, Auditory incongruent; L, left side; R, right side; B, both sides; and --, absence of a stimulus feature. Bold with * = *p* < 0.05.

False alarm rates to invalid stimuli were higher than error rates for target stimuli but remained relatively low overall. Friedman tests revealed no significant differences in false alarm rates across invalid stimulus conditions within any age group (all *p* ≥ 0.06). Two uncorrected Kruskal–Wallis tests reached significance, reflecting higher false alarm rates in both older groups than the <30yo group when the visual distractor and auditory stimulus were presented on opposite sides (invalid left distractor/right sound: H(2) = 7.40, *p* = 0.02, and invalid right distractor/left sound: H(2) = 9.46, *p* = 0.009). However, neither comparison remained significant following Šidák correction for multiple comparisons, indicating that overall accuracy was relatively comparable across age groups and stimulus conditions.

#### Motor responses for the visual discrimination task

To simplify interpretation and focus on the primary aims of the study, the main analyses were collapsed across target side because the overall pattern of multisensory facilitation and distractor-related costs was similar for left- and right-sided targets. Although target side produced some significant main and interaction effects, these were secondary to the study aims and are therefore presented in Supplementary Information D, together with the corresponding analyses of tap times.

**Figure 3:**
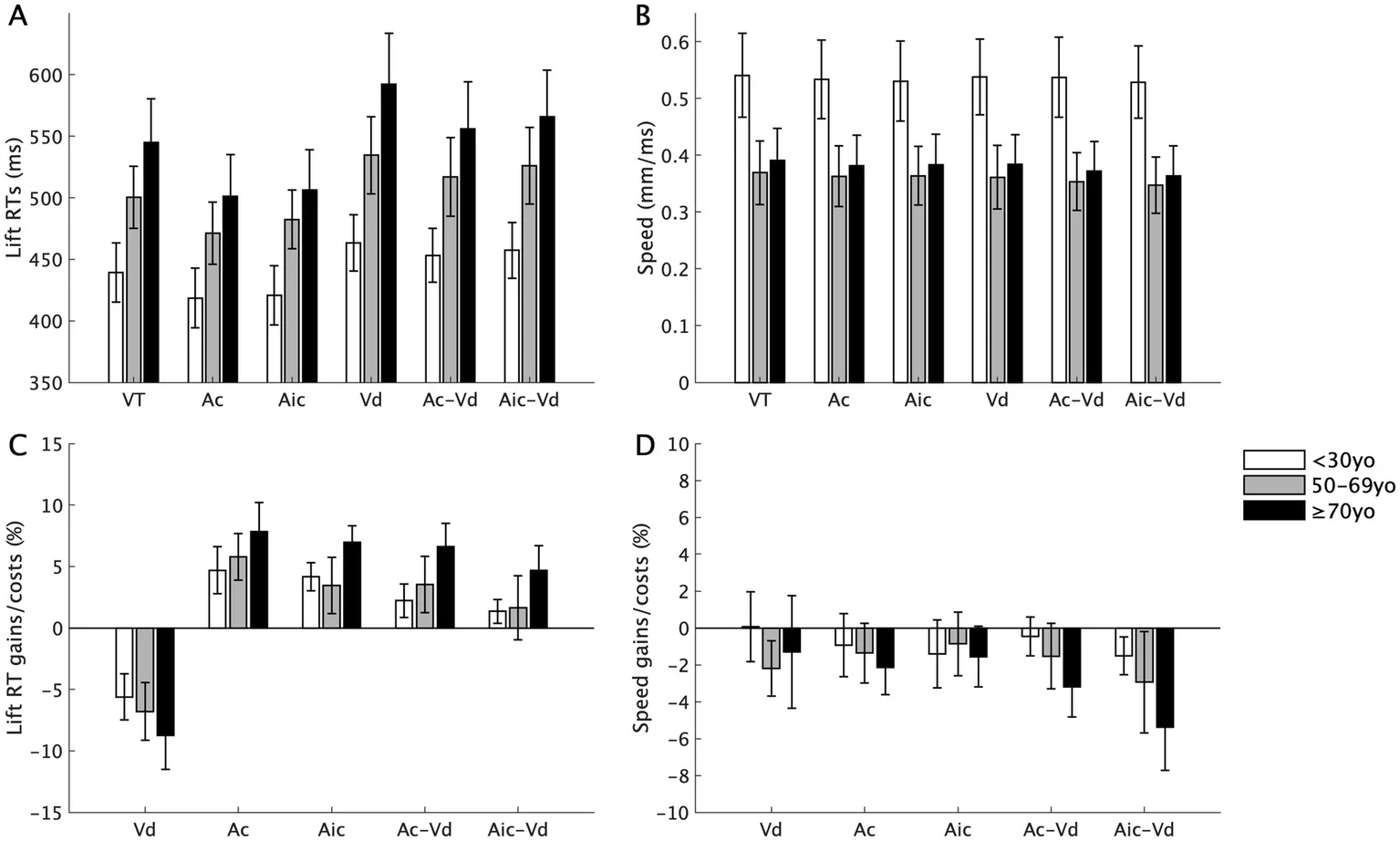
Motor responses on the visual discrimination task. Mean lift RTs **(A)** and movement speed **(B)** for visual targets (VT) presented with and without visual distractors (Vd), and with spatially congruent (Ac) and incongruent (Aic) auditory stimuli for each age group: <30yo, 50–69yo, and ≥70yo. Percentage change relative to the corresponding unisensory condition (i.e., Ac and Aic relative to VT, and Ac-Vd and Aic-Vd relative to Vd) are shown for lift RTs **(C)** and movement speed **(D)**. Positive values represent multisensory gains whereas negative values represent costs, calculated relative to VT or Vd. For the Vd condition, percentage change was calculated relative to the VT condition. Error bars represent 95% confidence intervals (CIs).

The fastest lift RTs were observed for multisensory stimuli with no visual distractor, yet speed was often fastest for the unisensory visual condition (see Figures 3A,B). Lift RTs and movement speed were analysed using a 2 (distractor: present, absent) × 3 (auditory stimulus: none, Ac, and Aic) × 3 (age group: <30yo, 50–69yo, and ≥70yo) mixed-design ANOVA.

For lift RTs, there were significant main effects of age group, *F*(2, 46) = 14.09, *p* < 0.001, partial *η*^2^ = 0.38, distractor, *F*(1, 46) = 147.54, *p* < 0.001, partial *η*^2^ = 0.76, and auditory stimulus, *F*(1.42, 65.25) = 89.84, *p* < 0.001, partial *η*^2^ = 0.66. Follow-up analyses showed that the <30yo group responded significantly faster than both the 50–69yo (*p* = 0.009) and ≥70yo (*p* < 0.001) groups, whereas the two older groups did not differ significantly (*p* = 0.15). Lift RTs were significantly slower in the presence of distractors (*p* < 0.001), and all three auditory conditions differed significantly from one another (all *p* < 0.001), with the fastest responses observed for spatially congruent auditory stimuli (Ac), followed by incongruent auditory stimuli (Aic), and the slowest responses for the visual-only condition. There were also significant Age Group × Distractor, *F*(2, 46) = 3.56, *p* = 0.04, partial *η*^2^ = 0.13, Age Group × Auditory Stimulus, *F*(2.84, 65.25) = 8.47, *p* < 0.001, partial *η*^2^ = 0.27, and Distractor × Auditory Stimulus interactions, *F*(1.92, 88.45) = 9.45, *p* < 0.001, partial *η*^2^ = 0.17, whereas the three-way interaction was not significant, *F*(3.85, 88.45) = 0.32, *p* = 0.86, partial *η*^2^ = 0.01. Follow-up simple effects analyses confirmed that distractors significantly slowed lift RTs in every age group and auditory condition (all *p* < 0.001). Likewise, audiovisual facilitation was evident across all age groups, with responses significantly faster for Ac than Aic and for Aic than the visual-only condition (all *p* ≤ 0.009). The significant interactions reflected differences in the magnitude, rather than the overall pattern, of these effects: distractor-related slowing increased with age, being greatest in the ≥70yo group, whereas the pattern of audiovisual facilitation remained consistent across all age groups.

For movement speed, there were significant main effects of age group, *F*(2, 46) = 11.89, *p* < 0.001, partial *η*^2^ = 0.34, distractor, *F*(1, 46) = 8.87, *p* = 0.005, partial *η*^2^ = 0.16, and auditory stimulus, *F*(1.52, 69.81) = 24.70, *p* < 0.001, partial *η*^2^ = 0.35. Follow-up analyses showed that the <30yo group moved significantly faster than both the 50–69yo and ≥70yo groups (both *p* < 0.001), whereas the two older groups did not differ significantly (*p* = 0.95). Movement speed was significantly slower in the presence of distractors (*p* = 0.005), and all three auditory conditions differed significantly from one another (all *p* ≤ 0.017), with the fastest movements observed for the visual-only (VT) condition, followed by the spatially congruent audiovisual (Ac) condition, and the slowest movements for the spatially incongruent audiovisual (Aic) condition. There was also a significant Distractor × Auditory Stimulus interaction, *F*(1.98, 91.04) = 4.18, *p* = 0.019, partial *η*^2^ = 0.08. Follow-up simple effects analyses confirmed that distractors significantly slowed movement speed in the Ac (*p* = 0.030) and Aic (*p* = 0.001) conditions, but not in the VT condition (*p* = 0.074). The interaction reflected differences in the magnitude of distractor-related slowing across stimulus conditions: in the absence of distractors, movement speed was slower for both audiovisual conditions than for the VT condition, with no difference between Ac and Aic, whereas in the presence of distractors all three conditions differed significantly, with movement speed slowing progressively from VT to Ac to Aic. No interactions involving age group were significant.

As can be observed in Figure 3B, absolute movement speed varied substantially across participants in all conditions. However, the relative percentage differences between the corresponding unisensory and multisensory conditions were considerably less variable across participants (Figure 3D). To account for this between-participant variability in absolute movement speed, percentage gains (or costs) relative to the corresponding unisensory condition were also examined.

#### Multisensory gains and distractor costs in the visual discrimination task

To facilitate comparisons across age groups and stimulus conditions while accounting for baseline differences in response speed, all measures were converted to percentage gains (or costs) relative to their corresponding unisensory condition (i.e., Ac and Aic were expressed relative to VT, whereas Ac-Vd and Aic-Vd were expressed relative to Vd). In addition, the percentage cost of the visual distractor was calculated by comparing the Vd condition with the corresponding VT condition. The formula was adjusted so that positive values always represented gains (i.e., faster motor responses relative to the corresponding unisensory condition), whereas negative values represented costs (i.e., slower motor responses relative to the corresponding unisensory condition). See Supplementary Information E for one-sample *t*-tests against zero assessing the significance of absolute gains within each group and condition. We used 5 (stimulus type: Ac, Aic, Vd, Ac-Vd, and Aic-Vd) × 3 (age group: <30yo, 50–69yo, and ≥70yo) mixed-design ANOVAs to examine multisensory and distractor-related gains and costs in lift RTs and movement speed.

As expected, all audiovisual conditions produced significant percentage gains for lift RTs relative to their corresponding unisensory condition, whereas the unisensory visual distractor (Vd) alone produced significant percentage costs (Figure 3C). There was a significant main effect of stimulus type, *F*(2.53, 116.29) = 146.90, *p* < 0.001, partial *η*^2^ = 0.76. The main effect of age group was also significant, *F*(2, 46) = 3.33, *p* = 0.045, partial *η*^2^ = 0.13, and the pattern of gains across stimulus types differed significantly between age groups, as reflected by a significant Age Group × Stimulus Type interaction, *F*(5.06, 116.29) = 4.64, *p* < 0.001, partial *η*^2^ = 0.17. Follow-up analyses indicated that age-related differences were confined to the distractor-related conditions. Specifically, the ≥70yo group exhibited significantly greater gains than the <30yo group for the Vd (*p* = 0.029), Ac-Vd (*p* = 0.002), and Aic-Vd (*p* = 0.04) conditions, and greater gains than the 50–69yo group for the Vd (*p* = 0.005) and Ac-Vd (*p* < 0.05) conditions. No significant age-group differences were observed for the Ac or Aic conditions. Across all three age groups, both the Ac and Aic conditions produced significantly greater gains than the Vd condition (all *p* < 0.001) and did not differ significantly from each other. The interaction reflected age-related differences in the magnitude of distractor-related gains rather than the overall pattern of multisensory facilitation: the effects of audiovisual spatial congruency were confined to the distractor conditions, with the Aic-Vd condition producing significantly smaller gains than the Ac condition in all three age groups (all *p* ≤ 0.003), whereas the Ac-Vd and Aic-Vd conditions differed significantly only in the ≥70yo group (*p* = 0.03). The percentage gains for taps followed a similar pattern to that observed for lift RTs (Supplementary Information F).

Overall, movement speed costs were greatest for visual targets presented with a distractor and an incongruent auditory stimulus (Aic-Vd; Figure 3D). There was a significant main effect of stimulus type, *F*(2.07, 95.18) = 3.62, *p* = 0.03, partial *η*^2^ = 0.07. The main effect of age group did not reach significance, *F*(2, 46) = 2.90, *p* = 0.07, partial *η*^2^ = 0.11, and the Stimulus Type × Age Group interaction was not significant, *F*(4.14, 95.18) = 1.31, *p* = 0.27, partial *η*^2^ = 0.05. Follow-up analyses of the significant main effect of stimulus type showed that movement speed costs were significantly greater for the Aic-Vd condition than for the Ac (*p* = 0.045), Aic (*p* = 0.032), and Ac-Vd (*p* = 0.009) conditions. No other pairwise comparisons reached significance.

**Figure 4:**
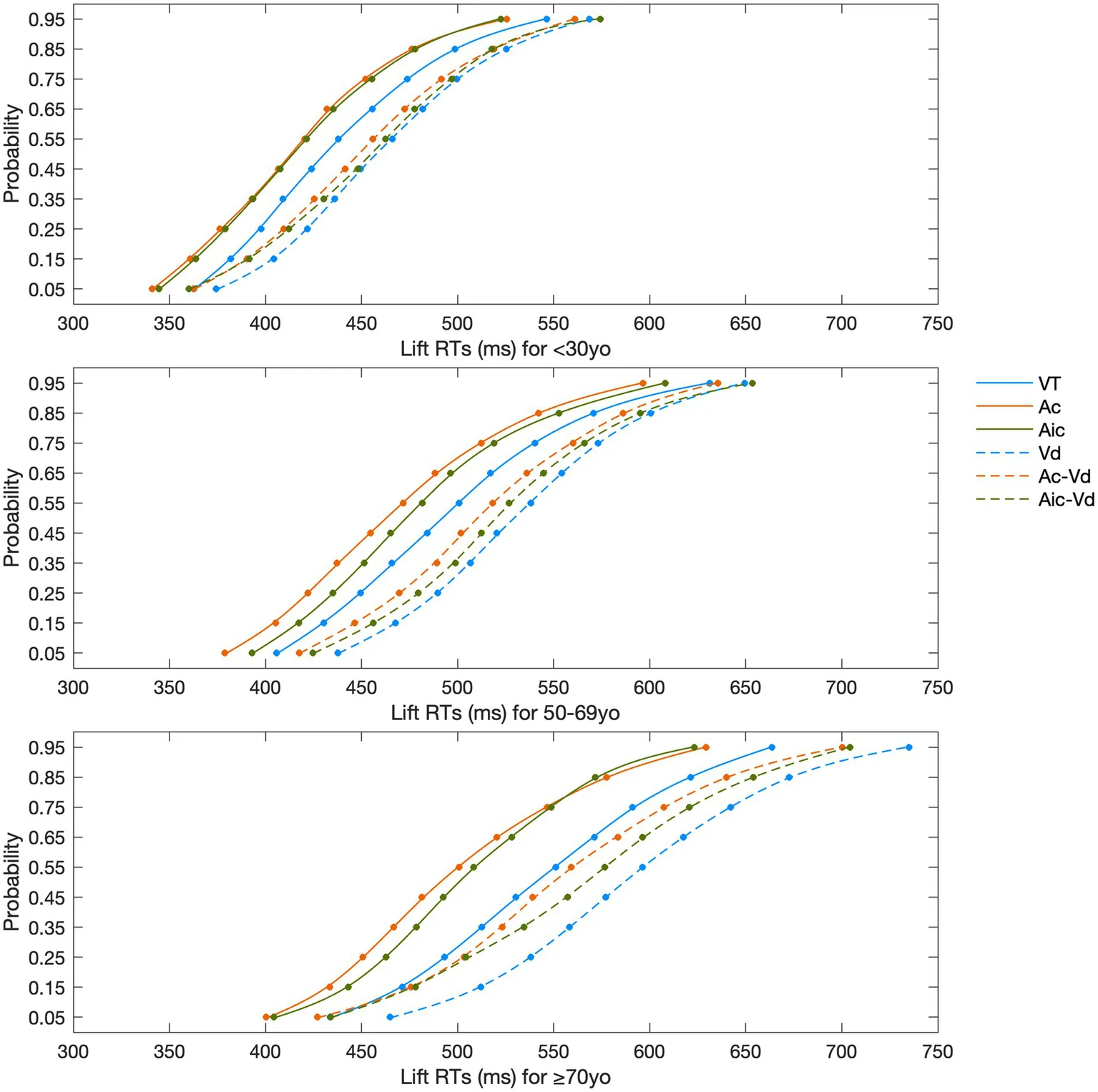
Cumulative density functions (CDFs) for lift RTs to visual targets in the discrimination task, collapsed across left- and right-sided target presentations. CDFs are shown for visual targets presented alone (VT) or combined with spatially congruent (Ac) or spatially incongruent (Aic) auditory stimuli, with and without a visual distractor (Vd), for each age group.

In the visual discrimination task, there were no redundant signals; therefore, Miller’s race-model test could not be applied. Nevertheless, we computed cumulative distribution functions (CDFs) for lift RTs. As shown in Figure 4, motor responses in the older adults were more variable. Consistent with the patterns observed in the multisensory detection task, the presence of an auditory stimulus shifted the entire distribution of lift RTs toward faster responses (leftward shift) relative to the unisensory conditions. In contrast, the presence of distractors caused the entire distribution of lift RTs to shift toward slower responses. Notably, an auditory stimulus that was spatially congruent with the visual target counteracted the distractor effect, most prominently in the 70yo group. Tap times mirrored the patterns we observed for lift RTs (see Supplementary Information F). Note that removing the left-handed participants from this analysis did not change the pattern of significant outcomes for multisensory gains and distractor related costs.

### Multisensory detection vs. visual discrimination analysis

**Figure 5:**
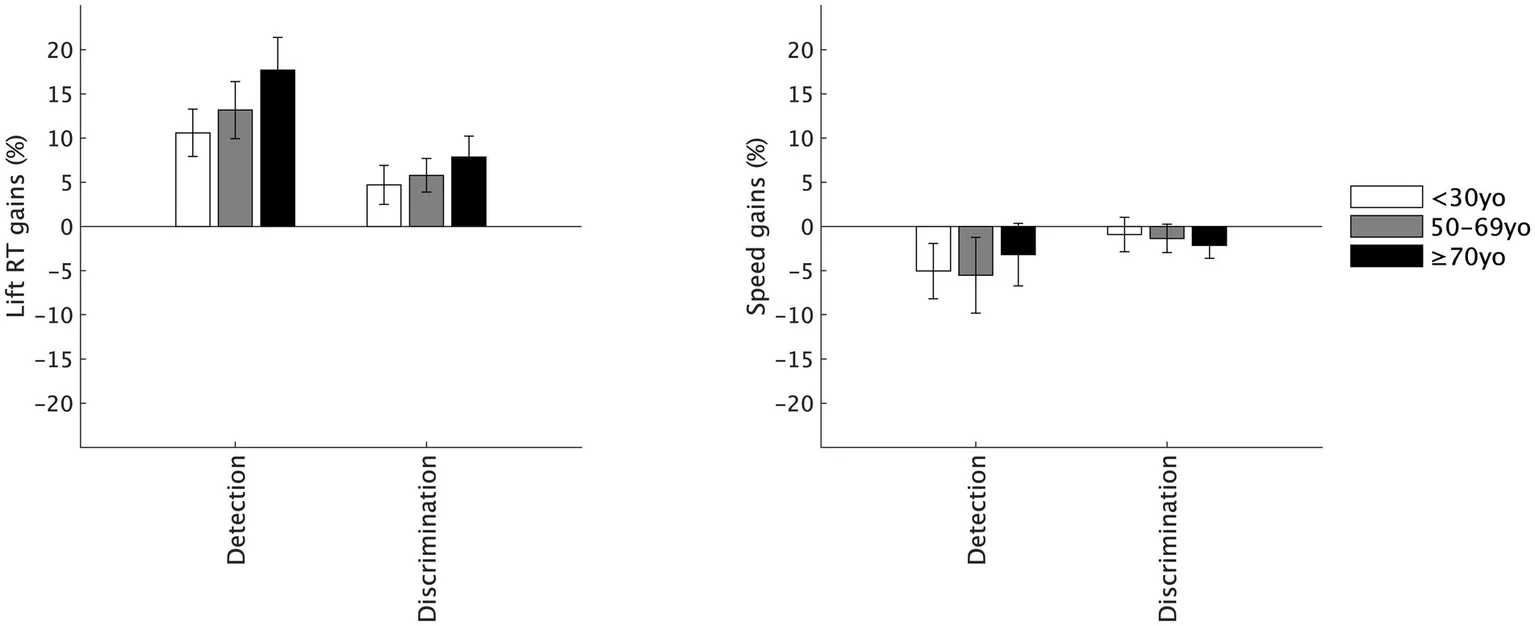
Percentage gains and costs relative to the faster of the unisensory targets in the detection and the visual discrimination task (i.e., this naturally being the visual target (VT) in the discrimination task) for Lift RTs and Speed for each age group. Error bars represent 95% CI.

Previous research has shown that multisensory facilitation is highly task dependent (Barutchu and Spence, 2021; Giray and Ulrich, 1993; Miller, 1982). We therefore compared percentage multisensory gains across the detection and visual discrimination tasks to determine whether task demands differentially influenced movement initiation (lift RTs) and execution (movement speed). Specifically, gains for the three measures in the detection task were compared with those for the spatially congruent audiovisual condition (Ac) in the visual discrimination task (Figure 5). A 2 (Task: detection, discrimination) × 2 (Measure: lift RT, movement speed) × 3 (Age group: <30 years, 50–69 years, ≥70 years) mixed ANOVA revealed significant main effects of task, *F*(1, 44) = 10.17, *p* = 0.003, partial *η*^2^ = 0.19, measure, *F*(1, 44) = 323.05, *p* < 0.001, partial *η*^2^ = 0.88, and age group, *F*(2, 44) = 3.92, *p* = 0.03, partial *η*^2^ = 0.15. Percentage multisensory gains were greater for the detection than the discrimination task. There was also a significant Measure × Age Group interaction, *F*(2, 44) = 3.96, *p* = 0.03, partial *η*^2^ = 0.15. Follow-up analyses showed that lift RT gains were greater than movement speed gains in all three age groups (all *p* < 0.001), with the difference between lift RT and movement speed gains increasing progressively with age. Across tasks and measures, the ≥70-year group exhibited greater percentage multisensory gains than both the <30-year (*p* = 0.03) and 50–69-year (*p* = 0.04) groups, whereas the <30-year and 50–69-year groups did not differ significantly (*p* = 0.88). The Task × Measure interaction was also significant, *F*(1, 44) = 46.73, *p* < 0.001, partial *η*^2^ = 0.52, reflecting lower multisensory gains in lift RTs and smaller movement-speed costs in the discrimination task compared with the detection task (all *p* < 0.005). Thus, both the facilitatory effects on movement initiation and the associated costs to movement speed were reduced in the discrimination task. The Task × Age Group interaction, *F*(2, 44) = 2.23, *p* = 0.12, partial *η*^2^ = 0.09, and the Task × Measure × Age Group interaction, *F*(2, 44) = 0.05, *p* = 0.95, partial *η*^2^ = 0.002, were not significant. Thus, the magnitude of multisensory facilitation differed across tasks and behavioural measures, but the overall pattern was consistent across age groups.

## Discussion

The results of the present study demonstrates that audiovisual stimuli robustly facilitate the initiation of goal-directed motor actions, as shown by faster lift RTs and tap RTs in both a simple detection task and a more demanding visual discrimination task. By separately examining movement initiation and movement speed, we found that improvements in movement initiation coincided with slower movement execution, with these movement-speed costs being more prominent in the faster simple detection task than in the visual discrimination task. Despite this reduction in movement speed, overall task performance, as reflected in tap completion and accuracy, remained facilitated, suggesting that multisensory integration may promote earlier movement initiation while preserving the accuracy of goal-directed actions. Visual distractors slowed motor responses more in older than in younger adults. Importantly, older adults exhibited significantly greater gains in lift RTs than younger adults in the detection task, even after controlling for general age-related slowing. However, this age-related multisensory advantage did not extend to the more complex visual discrimination task. Together, these findings confirm that multisensory facilitation is task-specific, primarily benefits the initiation of actions at a small cost to movement speed, and that both distractor costs and multisensory gains are amplified with age.

Although audiovisual stimuli facilitated both lift RTs and tap RTs, movement speed was reduced by approximately 5% in the detection task. In the visual discrimination task, movement speed costs were observed only in the older age groups under conditions of audiovisual distraction. As RTs were substantially slower in the visual discrimination task than in the detection task, these findings suggest that the influence of multisensory integration on movement execution is task- and context-dependent. Consequently, the multisensory gains observed at movement initiation were attenuated, but not eliminated, by the time participants completed the movement. This attenuation was evident in the tap RTs, which continued to show significant multisensory facilitation. Together, these findings suggest that the early facilitatory effects of multisensory integration on movement initiation may be followed by a later calibration phase during movement execution that refines movement precision. This two-stage process aligns with accounts of multisensory motor control that differentiate rapid initiation from subsequent corrective motor adjustments during execution and is consistent with mechanisms proposed in visual–haptic integration (see Camponogara, 2023 for review). Such adjustment is expected in tasks that place demands on both speed and accuracy. Importantly, these movement-speed costs were modest and were more than offset by the substantially larger facilitation of movement initiation, such that multisensory facilitation remained evident at tap completion, indicating that targets were reached more efficiently overall. Furthermore, error rates for target stimuli did not differ significantly across stimulus types or age groups in the detection task, providing no evidence of a speed-accuracy trade-off. Collectively, these findings suggest that multisensory integration enhances the initiation and overall execution of goal-directed actions without compromising accuracy, with only a modest, task- and context-dependent cost to movement speed.

Enhanced multisensory gains were observed in older adults in the audiovisual detection task but not in the discrimination task, a pattern partly consistent with studies reporting increased multisensory processing in older adults and in individuals with sensory loss (Brooks et al., 2018; Cerella, 1985; Frassinetti et al., 2005; Humes et al., 2013; Laurienti et al., 2006; Peter et al., 2019). Age-related sensory decline often reduces the fidelity of unisensory inputs, suggesting that diminished sensory reliability in ageing may amplify multisensory integration as a compensatory mechanism. Interestingly, no such enhancements were observed in the visual discrimination task. Multisensory facilitation is typically strongest under conditions with redundant signals, when attention is distributed across multiple sensory channels. In the detection task, both the auditory and visual signals conveyed task-relevant information and could therefore contribute to response initiation. In contrast, the auditory signal in the discrimination task was task irrelevant and non-predictive of target location and therefore needed to be ignored and could not be used to guide the response. This distinction was particularly important in the audiovisual incongruent condition, where the auditory signal was spatially congruent with the visual distractor and may have combined with it to form a multisensory distractor. Rather than facilitating target processing, the auditory signal may therefore have increased competition between the target and distractor. This competition may have been particularly disruptive in older adults, who were more susceptible to the task-irrelevant auditory signal. Greater attentional capture by the multisensory distractor would require disengagement and reorientation towards the target on the opposite side, consistent with the slower movement speeds observed in this condition. In addition, recent evidence indicates that switching between unisensory targets incurs greater costs than switching from multisensory targets, resulting in greater slowing for unisensory than for multisensory stimuli (Barutchu and Spence, 2020, 2021; Kreutzfeldt et al., 2015; Otto and Mamassian, 2012, 2016). As shown in the preliminary analyses in Supplementary Information C, older adults appear more affected by sensory switch costs, which may have further contributed to the enhanced multisensory facilitation in the detection task and its absence in the visual discrimination task. In the discrimination task, attention was focused solely on the visual modality, and the auditory signal was task-irrelevant and non-predictive of the target’s location, eliminating switching between sensory targets. Importantly, the enhanced multisensory gains observed in older adults are unlikely to be attributable to response bias (Holmes, 2007, 2009) as accuracy, and thus detectability, did not differ between unisensory and multisensory conditions in the detection task, and the entire RT distribution was faster for multisensory compared to unisensory stimuli in both tasks. Taken together, these results suggest that enhanced multisensory gains in older adults are task-dependent and may reflect a mechanism that helps to protect against the costs associated with sensory switching.

In the visual discrimination task, and in the absence of distractors, audiovisual facilitation of object discrimination was evident regardless of the spatial congruence of the auditory signal. The relatively small active display of the touchscreen may have kept all stimuli within the audiovisual spatiotemporal window for multisensory integration. This result also aligns with evidence that when spatial judgments are not required, the spatial alignment of sensory inputs plays a limited role (Spence, 2013). Instead, temporal synchrony appeared to be the primary driver of facilitation, in line with accounts of the “pip-and-pop” effect (Van der Burg et al., 2008), and studies that show both spatially and semantically irrelevant auditory signals can enhance visual object identification and discrimination (e.g., Barutchu et al., 2013; Hillyard et al., 2016; McDonald et al., 2000; Spence and Ngo, 2012). Furthermore, given the visual system’s higher spatial resolution and, in turn, spatial reliability, the visual target likely dominated over audition, thus capturing the perceived location of the auditory stimulus for binding, as in the ventriloquist effect (Alais and Burr, 2004). In the absence of visual distractors, auditory spatial congruency had little effect. However, when visual distractors were present, spatially congruent auditory signals conferred an additional benefit, suggesting that auditory spatial information becomes increasingly important under conditions of visual competition.

As expected, the presence of visual distractors significantly impaired performance, slowing lift RTs, an effect that likely reflects the increased attentional demands imposed by distractors under conditions of relatively low perceptual load (Lavie, 1995; Tsal and Benoni, 2010). Distractor costs were greater in older adults, consistent with age-related declines in selective attention that allow attention to spread more broadly and increase the susceptibility to distraction. All adults benefited from spatially congruent audiovisual stimuli in the presence of distractors; however, older adults were significantly more affected by the spatial congruence of auditory stimuli under these conditions, suggesting that spatially aligned audiovisual signals may help counteract age-related vulnerability to distraction, even when tested on small portable devices. The different senses are thought to recruit partially distinct, but nevertheless highly interconnected pools of attentional resources (Brand-D'Abrescia and Lavie, 2008; Matusz et al., 2015; Spence and Ho, 2008; Tellinghuisen and Nowak, 2003), and under multisensory conditions these resources may combine to reorient attention across modalities and toward the target location, thereby mitigating distractor interference. Multisensory processes are calibrated by the predictability and reliability of individual sensory signals (Alais and Burr, 2004; Spence et al., 2001; Zuanazzi and Noppeney, 2019); thus audiovisual spatial congruence may be especially relevant in older adults, whose auditory and visual systems are more likely to be degraded and less reliable (Humes et al., 2013; Park and Reuter-Lorenz, 2009) effectively increasing perceptual load even with a single competing distractor, narrowing the spatial focus of multisensory attention, and optimizing the integration of spatially congruent audiovisual signals. As with attention, the spatiotemporal focus of multisensory integration may narrow with increasing task difficulty. Reduced sensory sensitivity and the requirement to make goal-directed actions to the target’s location may have further amplified the effect of auditory congruence. Taken together, these findings suggest that the spatial “rule” of multisensory integration plays a critical role in helping, particularly older adults, to mitigate distractor costs, thus highlighting the importance of considering spatial congruence in multisensory research on ageing.

A robust lateralised motor bias was also observed in the visual discrimination task at the initiation of actions; Participants consistently responded more rapidly to visual targets presented on the right side of the screen than to those on the left. This right-field advantage did not alter the direction of multisensory gains or distractor-related costs. Such lateralised effects are typically absent in traditional button- or keypress-based paradigms (e.g., Hillyard et al., 2016; Spence and Driver, 1997), which generally provide a single measure of response latency. In contrast, the touchscreen paradigm allowed movement initiation to be quantified separately from movement execution, providing greater sensitivity to identify stage-specific sensorimotor effects. Consistent with Daini et al. (2018), preliminary follow-up analyses indicated that this bias may reverse in left-handed individuals (Supplementary Information D). In the present study, participants were free to use their preferred hand. Notably, the right-field advantage was evident across all motor measures, including the initial lift RT, indicating that it cannot be attributed solely to movement direction or distance (for example, reaching across versus to the side of the body) and may instead reflect lateralised differences in sensorimotor preparatory processes. Importantly, excluding left-handed participants from the analysis did not change the overall pattern of multisensory gains or distractor-related costs.

Several limitations should be considered when interpreting the present findings. Movement speed was estimated from the straight-line distance between the docking position and tap location, providing an endpoint-to-endpoint average speed rather than the actual speed along the movement trajectory. Consequently, the touchscreen platform could not capture continuous finger trajectories or online movement corrections, which may be quantified using motion-tracking systems in future studies. The discrimination task was also intentionally designed to be relatively easy so that participants across the adult lifespan could perform it reliably. Consequently, response accuracy remained high across all age groups, limiting the influence of visual distractors on accuracy. It is therefore likely that greater task demands would reveal larger distractor-related effects, particularly in older adults, and further alter the influence of multisensory integration on motor responses. In addition, although participants were stratified into predefined age bands to examine potential stage-based changes across later adulthood, larger studies treating age as a continuous variable are needed to determine whether multisensory processing changes progressively across the adult lifespan. Finally, vision and hearing status were determined by self-report rather than objective clinical assessment. Future studies should incorporate standardised measures of sensory function to better understand how sensory acuity influences multisensory integration across the adult lifespan.

In conclusion, these findings extend current theories of multisensory integration and action control by demonstrating that multisensory benefits emerge most robustly during early processing, prior to the initiation of movement. This pattern aligns with theoretical accounts proposing that audiovisual integration primarily supports early sensory processing and motor planning rather than late-stage motor refinement. The accompanying modest slowing of subsequent movement suggests that initial facilitatory gains are followed by compensatory adjustments to maintain the precision of goal-directed actions. Age-related patterns indicate that older adults show enhanced multisensory benefits during simple detection, but greater distractor interference and sensitivity to spatial incongruence during discrimination. This is consistent with theoretical accounts in attention, proposing that the spatial window of audiovisual integration narrows under attentional load to prioritise goal-relevant information. More broadly, the results reinforce the view that multisensory integration is dynamically tuned by attentional demands and environmental uncertainty, rather than operating as a fixed sensory gain. Beyond their theoretical implications, the robustness of these effects across age groups underscores the translational potential of portable touchscreen paradigms for accessible assessment and intervention in everyday environments, including educational, workplace, and clinical rehabilitation settings.

## Data Availability

The raw data supporting the conclusions of this article will be made available by the authors, without undue reservation.

## References

[ref1] Ahmed F. Nidiffer A. R. Lalor E. C. (2023a). The effect of gaze on EEG measures of multisensory integration in a cocktail party scenario. Front. Hum. Neurosci. 17:1283206. doi: 10.3389/fnhum.2023.1283206, 38162285 PMC10754997

[ref2] Ahmed F. Nidiffer A. R. O'Sullivan A. E. Zuk N. J. Lalor E. C. (2023b). The integration of continuous audio and visual speech in a cocktail-party environment depends on attention. NeuroImage 274:120143. doi: 10.1016/j.neuroimage.2023.120143, 37121375 PMC12956257

[ref3] Alain C. Woods D. L. (1999). Age-related changes in processing auditory stimuli during visual attention: evidence for deficits in inhibitory control and sensory memory. Psychol. Aging 14, 507–519. doi: 10.1037//0882-7974.14.3.507, 10509703

[ref4] Alais D. Burr D. (2004). The ventriloquist effect results from near-optimal bimodal integration. Curr. Biol. 14, 257–262. doi: 10.1016/j.cub.2004.01.029, 14761661

[ref5] Atkin C. Stacey J. E. Allen H. A. Henshaw H. Roberts K. L. Badham S. P. (2024). Older adults do not show enhanced benefits from multisensory information on speeded perceptual discrimination tasks. Neurobiol. Aging 142, 65–72. doi: 10.1016/j.neurobiolaging.2024.08.003, 39173227

[ref6] Barutchu A. Crewther D. P. Crewther S. G. (2009). The race that precedes coactivation: development of multisensory facilitation in children. Dev. Sci. 12, 464–473. doi: 10.1111/j.1467-7687.2008.00782.x, 19371371

[ref7] Barutchu A. Crewther S. G. Fifer J. Shivdasani M. N. Innes-Brown H. Toohey S. . (2011). The relationship between multisensory integration and IQ in children. Dev. Psychol. 47, 877–885. doi: 10.1037/a0021903, 21142364

[ref8] Barutchu A. Fifer J. M. Shivdasani M. N. Crewther S. G. Paolini A. G. (2020). The interplay between multisensory associative learning and IQ in children. Child Dev. 91, 620–637. doi: 10.1111/cdev.13210, 30620403

[ref9] Barutchu A. Freestone D. R. Innes-Brown H. Crewther D. P. Crewther S. G. (2013). Evidence for enhanced multisensory facilitation with stimulus relevance: an electrophysiological investigation. PLoS One 8:e52978. doi: 10.1371/journal.pone.0052978, 23372652 PMC3553102

[ref10] Barutchu A. Spence C. (2020). An experimenter's influence on motor enhancements: the effects of letter congruency and sensory switch-costs on multisensory integration. Front. Psychol. 11:588343. doi: 10.3389/fpsyg.2020.588343, 33335500 PMC7736551

[ref11] Barutchu A. Spence C. (2021). Top-down task-specific determinants of multisensory motor reaction time enhancements and sensory switch costs. Exp. Brain Res. 239, 1021–1034. doi: 10.1007/s00221-020-06014-3, 33515085 PMC7943519

[ref12] Barutchu A. Toohey S. Shivdasani M. N. Fifer J. M. Crewther S. G. Grayden D. B. . (2019). Multisensory perception and attention in school-age children. J. Exp. Child Psychol. 180, 141–155. doi: 10.1016/j.jecp.2018.11.021, 30655099

[ref13] Becker P. T. (2010). Taking note of trends in age groupings. Res. Nurs. Health 33, 475–476. doi: 10.1002/nur.20401, 20890949

[ref14] Bischoff M. Zentgraf K. Pilgramm S. Stark R. Kruger B. Munzert J. (2014). Anticipating action effects recruits audiovisual movement representations in the ventral premotor cortex. Brain Cogn. 92, 39–47. doi: 10.1016/j.bandc.2014.09.010, 25463138

[ref15] Bolognini N. Senna I. Maravita A. Pascual-Leone A. Merabet L. B. (2010). Auditory enhancement of visual phosphene perception: the effect of temporal and spatial factors and of stimulus intensity. Neurosci. Lett. 477, 109–114. doi: 10.1016/j.neulet.2010.04.044, 20430065 PMC3538364

[ref16] Brand-D'Abrescia M. Lavie N. (2008). Task coordination between and within sensory modalities: effects on distraction. Percept. Psychophys. 70, 508–515. doi: 10.3758/pp.70.3.508, 18459262

[ref17] Brooks C. J. Chan Y. M. Anderson A. J. McKendrick A. M. (2018). Audiovisual temporal perception in aging: the role of multisensory integration and age-related sensory loss. Front. Hum. Neurosci. 12:192. doi: 10.3389/fnhum.2018.00192, 29867415 PMC5954093

[ref18] Brunetti R. Indraccolo A. Mastroberardino S. Spence C. Santangelo V. (2017). The impact of cross-modal correspondences on working memory performance. J. Exp. Psycholol. Hum. Percept. Perform. 43, 819–831. doi: 10.1037/xhp0000348, 28345948

[ref19] Bruns P. Roder B. (2023). Development and experience-dependence of multisensory spatial processing. Trends Cogn. Sci. 27, 961–973. doi: 10.1016/j.tics.2023.04.012, 37208286

[ref20] Buchan J. N. Munhall K. G. (2011). The influence of selective attention to auditory and visual speech on the integration of audiovisual speech information. Perception 40, 1164–1182. doi: 10.1068/p6939, 22308887

[ref21] Camponogara I. (2023). The integration of action-oriented multisensory information from target and limb within the movement planning and execution. Neurosci. Biobehav. Rev. 151:105228. doi: 10.1016/j.neubiorev.2023.105228, 37201591

[ref22] Cerella J. (1985). Information processing rates in the elderly. Psychol. Bull. 98, 67–83. doi: 10.1037/0033-2909.98.1.67, 4034819

[ref23] Cienkowski K. M. Carney A. E. (2002). Auditory-visual speech perception and aging. Ear Hear. 23, 439–449. doi: 10.1097/00003446-200210000-00006, 12411777

[ref24] Couth S. Gowen E. Poliakoff E. (2018). Using race model violation to explore multisensory responses in older adults: enhanced multisensory integration or slower unisensory processing? Multisens. Res. 31, 151–174. doi: 10.1163/22134808-00002588, 31264629

[ref25] Daini R. Vallar G. Arduino L. S. (2018). Why we move to the right? The dominant hand motor-spatial bias. J. Exp. Psychol. Gen. 147, 1488–1502. doi: 10.1037/xge0000476, 30272464

[ref26] de Dieuleveult A. L. Siemonsma P. C. van Erp J. B. Brouwer A. M. (2017). Effects of aging in multisensory integration: a systematic review. Front. Aging Neurosci. 9:80. doi: 10.3389/fnagi.2017.00080 PMC5368230 28400727

[ref27] Diederich A. Colonius H. (2015). The time window of multisensory integration: relating reaction times and judgments of temporal order. Psychol. Rev. 122, 232–241. doi: 10.1037/a0038696, 25706404

[ref28] Downing H. C. Barutchu A. Crewther S. G. (2014). Developmental trends in the facilitation of multisensory objects with distractors. Front. Psychol. 5:1559. doi: 10.3389/fpsyg.2014.01559, 25653630 PMC4298743

[ref29] Doyle M. C. Snowden R. J. (2001). Identification of visual stimuli is improved by accompanying auditory stimuli: the role of eye movements and sound location. Perception 30, 795–810. doi: 10.1068/p3126, 11515953

[ref30] Driver J. Spence C. (1998). Cross-modal links in spatial attention. Philos. Trans. R. Soc. Lond. B Biol. Sci. 353, 1319–1331. doi: 10.1098/rstb.1998.0286, 9770225 PMC1692335

[ref31] Duncan J. (1980). The locus of interference in the perception of simultaneous stimuli. Psychol. Rev. 87, 272–300. doi: 10.1037/0033-295X.87.3.272, 7384344

[ref32] Ernst M. O. Banks M. S. (2002). Humans integrate visual and haptic information in a statistically optimal fashion. Nature 415, 429–433. doi: 10.1038/415429a, 11807554

[ref33] Fairhall S. L. Macaluso E. (2009). Spatial attention can modulate audiovisual integration at multiple cortical and subcortical sites. Eur. J. Neurosci. 29, 1247–1257. doi: 10.1111/j.1460-9568.2009.06688.x, 19302160

[ref34] Faul F. Erdfelder E. Lang A. G. Buchner A. (2007). G*power 3: a flexible statistical power analysis program for the social, behavioral, and biomedical sciences. Behav. Res. Methods 39, 175–191. doi: 10.3758/bf03193146, 17695343

[ref35] Frassinetti F. Bolognini N. Bottari D. Bonora A. Ladavas E. (2005). Audiovisual integration in patients with visual deficit. J. Cogn. Neurosci. 17, 1442–1452. doi: 10.1162/0898929054985446, 16197697

[ref36] Giray M. Ulrich R. (1993). Motor coactivation revealed by response force in divided and focused attention. J. Exp. Psychol. Hum. Percept. Perform. 19, 1278–1291. doi: 10.1037/0096-1523.19.6.1278, 8294892

[ref37] Gondan M. (2009). Testing the race model inequality in redundant stimuli with variable onset asynchrony. J. Exp. Psychol. Hum. Percept. Perform. 35, 575–579. doi: 10.1037/a0013620, 19331509

[ref38] Guerreiro M. J. Murphy D. R. Van Gerven P. W. (2010). The role of sensory modality in age-related distraction: a critical review and a renewed view. Psychol. Bull. 136, 975–1022. doi: 10.1037/a0020731, 21038938

[ref39] Hillyard S. A. Stormer V. S. Feng W. Martinez A. McDonald J. J. (2016). Cross-modal orienting of visual attention. Neuropsychologia 83, 170–178. doi: 10.1016/j.neuropsychologia.2015.06.003, 26072092

[ref40] Holmes N. P. (2007). The law of inverse effectiveness in neurons and behaviour: multisensory integration versus normal variability. Neuropsychologia 45, 3340–3345. doi: 10.1016/j.neuropsychologia.2007.05.025, 17663007

[ref41] Holmes N. P. (2009). The principle of inverse effectiveness in multisensory integration: some statistical considerations. Brain Topogr. 21, 168–176. doi: 10.1007/s10548-009-0097-2, 19404728

[ref42] Huang S. Li Y. Zhang W. Zhang B. Liu X. Mo L. . (2015). Multisensory competition is modulated by sensory pathway interactions with fronto-sensorimotor and default-mode network regions. J. Neurosci. 35, 9064–9077. doi: 10.1523/JNEUROSCI.3760-14.2015, 26085631 PMC6605163

[ref43] Humes L. E. Busey T. A. Craig J. Kewley-Port D. (2013). Are age-related changes in cognitive function driven by age-related changes in sensory processing? Atten. Percept. Psychophys. 75, 508–524. doi: 10.3758/s13414-012-0406-9, 23254452 PMC3617348

[ref44] Jia S. Zhang T. Zuo R. Xu B. (2023). Explaining cocktail party effect and McGurk effect with a spiking neural network improved by motif-topology. Front. Neurosci. 17:1132269. doi: 10.3389/fnins.2023.1132269, 37021133 PMC10067589

[ref45] Jones S. A. Noppeney U. (2021). Ageing and multisensory integration: a review of the evidence, and a computational perspective. Cortex 138, 1–23. doi: 10.1016/j.cortex.2021.02.001, 33676086

[ref46] Klapetek A. Ngo M. K. Spence C. (2012). Does crossmodal correspondence modulate the facilitatory effect of auditory cues on visual search? Atten. Percept. Psychophys. 74, 1154–1167. doi: 10.3758/s13414-012-0317-9, 22648604

[ref47] Kreutzfeldt M. Stephan D. N. Sturm W. Willmes K. Koch I. (2015). The role of crossmodal competition and dimensional overlap in crossmodal attention switching. Acta Psychol. 155, 67–76. doi: 10.1016/j.actpsy.2014.12.006, 25577489

[ref48] Kreyenmeier P. Bhuiyan I. Gian M. Chow H. M. Spering M. (2024). Smooth pursuit inhibition reveals audiovisual enhancement of fast movement control. J. Vis. 24:3. doi: 10.1167/jov.24.4.3, 38558158 PMC10996987

[ref49] Laurienti P. J. Burdette J. H. Maldjian J. A. Wallace M. T. (2006). Enhanced multisensory integration in older adults. Neurobiol. Aging 27, 1155–1163. doi: 10.1016/j.neurobiolaging.2005.05.024, 16039016

[ref50] Lavie N. (1995). Perceptual load as a necessary condition for selective attention. J. Exp. Psychol. Hum. Percept. Perform. 21, 451–468. doi: 10.1037//0096-1523.21.3.451, 7790827

[ref51] Levy J. Pashler H. Boer E. (2006). Central interference in driving: is there any stopping the psychological refractory period? Psychol. Sci. 17, 228–235. doi: 10.1111/j.1467-9280.2006.01690.x, 16507063

[ref52] Lovelace C. T. Stein B. E. Wallace M. T. (2003). An irrelevant light enhances auditory detection in humans: a psychophysical analysis of multisensory integration in stimulus detection. Brain Res. Cogn. Brain Res. 17, 447–453. doi: 10.1016/s0926-6410(03)00160-5, 12880914

[ref53] Mahoney J. R. Verghese J. Dumas K. Wang C. Holtzer R. (2012). The effect of multisensory cues on attention in aging. Brain Res. 1472, 63–73. doi: 10.1016/j.brainres.2012.07.014, 22820295 PMC3592377

[ref54] Malouka S. Loria T. Crainic V. Thaut M. H. Tremblay L. (2023). Auditory cueing facilitates temporospatial accuracy of sequential movements. Hum. Mov. Sci. 89:103087. doi: 10.1016/j.humov.2023.103087, 37060619

[ref55] Matusz P. J. Broadbent H. Ferrari J. Forrest B. Merkley R. Scerif G. (2015). Multi-modal distraction: insights from children's limited attention. Cognition 136, 156–165. doi: 10.1016/j.cognition.2014.11.031, 25497524

[ref56] McDonald J. J. Teder-Salejarvi W. A. Hillyard S. A. (2000). Involuntary orienting to sound improves visual perception. Nature 407, 906–908. doi: 10.1038/35038085, 11057669

[ref57] Miller J. (1982). Divided attention: evidence for coactivation with redundant signals. Cogn. Psychol. 14, 247–279. doi: 10.1016/0010-0285(82)90010-X, 7083803

[ref58] Miller J. (1986). Timecourse of coactivation in bimodal divided attention. Percept. Psychophys. 40, 331–343. doi: 10.3758/BF03203025, 3786102

[ref59] Miller J. (2016). Statistical facilitation and the redundant signals effect: what are race and coactivation models? Atten. Percept. Psychophys. 78, 516–519. doi: 10.3758/s13414-015-1017-z, 26555650

[ref60] Molholm S. Ritter W. Javitt D. C. Foxe J. J. (2004). Multisensory visual-auditory object recognition in humans: a high-density electrical mapping study. Cereb. Cortex 14, 452–465. doi: 10.1093/cercor/bhh007, 15028649

[ref61] Mozolic J. L. Hugenschmidt C. E. Peiffer A. M. Laurienti P. J. (2012). “Multisensory integration and aging,” in The Neural Bases of Multisensory Processes, eds. Murray M. M. Wallace M. T. (Boca Raton, FL: CRC Press/Taylor & Francis). 22593868

[ref62] Murakami T. Abe M. Wiratman W. Fujiwara J. Okamoto M. Mizuochi-Endo T. . (2018). The motor network reduces multisensory illusory perception. J. Neurosci. 38, 9679–9688. doi: 10.1523/JNEUROSCI.3650-17.2018, 30249803 PMC6595990

[ref63] National Academies of Sciences (2015). Cognitive Aging: Progress in Understanding and Opportunities for action. Washington, DC: The National Academies Press. 25879131

[ref64] Odgaard E. C. Arieh Y. Marks L. E. (2003). Cross-modal enhancement of perceived brightness: sensory interaction versus response bias. Percept. Psychophys. 65, 123–132. doi: 10.3758/bf03194789, 12699315

[ref65] Otto T. U. Mamassian P. (2012). Noise and correlations in parallel perceptual decision making. Curr. Biol. 22, 1391–1396. doi: 10.1016/j.cub.2012.05.031, 22771043

[ref66] Otto T. U. Mamassian P. (2016). Multisensory decisions: the test of a race model, its logic, and power. Multisens. Res. 30, 1–24. doi: 10.1163/22134808-00002541

[ref67] Park D. C. Reuter-Lorenz P. (2009). The adaptive brain: aging and neurocognitive scaffolding. Annu. Rev. Psychol. 60, 173–196. doi: 10.1146/annurev.psych.59.103006.093656, 19035823 PMC3359129

[ref68] Peiffer A. M. Mozolic J. L. Hugenschmidt C. E. Laurienti P. J. (2007). Age-related multisensory enhancement in a simple audiovisual detection task. Neuroreport 18, 1077–1081. doi: 10.1097/WNR.0b013e3281e72ae7, 17558300

[ref69] Peter M. G. Porada D. K. Regenbogen C. Olsson M. J. Lundstrom J. N. (2019). Sensory loss enhances multisensory integration performance. Cortex 120, 116–130. doi: 10.1016/j.cortex.2019.06.003, 31299497

[ref70] Peters C. M. Glazebrook C. M. (2020). Rhythmic auditory stimuli heard before and during a reaching movement elicit performance improvements in both temporal and spatial movement parameters. Acta Psychol. 207:103086. doi: 10.1016/j.actpsy.2020.103086, 32422419

[ref71] Raab D. H. (1962). Statistical facilitation of simple reaction times. Trans. N. Y. Acad. Sci. 24, 574–590. doi: 10.1111/j.2164-0947.1962.tb01433.x, 14489538

[ref72] Rey-Mermet A. Gade M. (2018). Inhibition in aging: what is preserved? What declines? A meta-analysis. Psychon. Bull. Rev. 25, 1695–1716. doi: 10.3758/s13423-017-1384-7, 29019064

[ref73] Rodrigues E. A. Djiberou Mahamadou A. J. Moreno S. (2025). The impact of lifestyle factors on trajectories of cognitive subtypes in the older adult population. Sci. Rep. 15:31744. doi: 10.1038/s41598-025-91171-0, 40877588 PMC12394718

[ref74] Roudaia E. Calabro F. J. Vaina L. M. Newell F. N. (2018). Aging impairs audiovisual facilitation of object motion within self-motion. Multisens. Res. 31, 251–272. doi: 10.1163/22134808-00002600, 31264625

[ref75] Salthouse T. A. (2009). When does age-related cognitive decline begin? Neurobiol. Aging 30, 507–514. doi: 10.1016/j.neurobiolaging.2008.09.023, 19231028 PMC2683339

[ref76] Schulte A. Thiel C. M. Gieseler A. Tahden M. Colonius H. Rosemann S. (2020). Reduced resting state functional connectivity with increasing age-related hearing loss and McGurk susceptibility. Sci. Rep. 10:16987. doi: 10.1038/s41598-020-74012-0, 33046800 PMC7550565

[ref77] Seitz A. R. Kim R. Shams L. (2006). Sound facilitates visual learning. Curr. Biol. 16, 1422–1427. doi: 10.1016/j.cub.2006.05.048, 16860741

[ref78] Sinnett S. Soto-Faraco S. Spence C. (2008). The co-occurrence of multisensory competition and facilitation. Acta Psychol. 128, 153–161. doi: 10.1016/j.actpsy.2007.12.002, 18207117

[ref79] Spence C. (2013). Just how important is spatial coincidence to multisensory integration? Evaluating the spatial rule. Ann. N. Y. Acad. Sci. 1296, 31–49. doi: 10.1111/nyas.12121, 23710729

[ref80] Spence C. (2025). Reflecting on the merging of the senses: a cognitive psychology perspective. Multisens. Res. 38, 231–253. doi: 10.1163/22134808-bja10139, 41167242

[ref81] Spence C. Driver J. (1996). Audiovisual links in endogenous covert spatial attention. J. Exp. Psychol. Hum. Percept. Perform. 22, 1005–1030. doi: 10.1037/0096-1523.22.4.1005, 8756965

[ref82] Spence C. Driver J. (1997). Audiovisual links in exogenous covert spatial orienting. Percept. Psychophys. 59, 1–22. doi: 10.3758/bf03206843, 9038403

[ref83] Spence C. Driver J. (2004). (eds.) Crossmodal space and crossmodal attention. Oxford, UK: Oxford University Press.

[ref84] Spence C. Ho C. (2008). “Crossmodal information processing in driving,” in Human Factors of visual Performance in Driving, ed. Castro C. (Boca Raton, FL: CRC Press), 187–200.

[ref85] Spence C. Ngo M. K. (2012). “Does attention or multisensory integration explain the crossmodal facilitation of masked visual target identification?” in The new Handbook of Multisensory Processing, ed. Stein B. E. (Cambridge, MA: MIT Press).

[ref86] Spence C. Nicholls M. E. Driver J. (2001). The cost of expecting events in the wrong sensory modality. Percept. Psychophys. 63, 330–336. doi: 10.3758/BF03194473, 11281107

[ref9001] Spence C. (2018). Multisensory perception. Stevens’ handbook of experimental psychology and cognitive neuroscience: Sensation, perception, and attention. In: J. T. Wixted (Ed.-in-Chief) and J. Serences (Vol. Ed.), 4th Edn. Sensation, perception, and attention. John Wiley & Sons. Vol. 2, p. 1–56., 23710729

[ref87] Stein B. E. London N. Wilkinson L. K. Price D. D. (1996). Enhancement of perceived visual intensity by auditory stimuli: a psychophysical analysis. J. Cogn. Neurosci. 8, 497–506. doi: 10.1162/jocn.1996.8.6.497, 23961981

[ref88] Stein B. E. Meredith M. A. (1993). The Merging of the Senses. Cambridge, MA: MIT Press.

[ref89] Stein B. E. Stanford T. R. (2008). Multisensory integration: current issues from the perspective of the single neuron. Nat. Rev. Neurosci. 9, 255–266. doi: 10.1038/nrn2331, 18354398

[ref90] Stevenson R. A. Fister J. K. Barnett Z. P. Nidiffer A. R. Wallace M. T. (2012). Interactions between the spatial and temporal stimulus factors that influence multisensory integration in human performance. Exp. Brain Res. 219, 121–137. doi: 10.1007/s00221-012-3072-1, 22447249 PMC3526341

[ref91] Talsma D. Doty T. J. Woldorff M. G. (2007). Selective attention and audiovisual integration: is attending to both modalities a prerequisite for early integration? Cereb. Cortex 17, 679–690. doi: 10.1093/cercor/bhk016, 16707740

[ref92] Talsma D. Senkowski D. Soto-Faraco S. Woldorff M. G. (2010). The multifaceted interplay between attention and multisensory integration. Trends Cogn. Sci. 14, 400–410. doi: 10.1016/j.tics.2010.06.008, 20675182 PMC3306770

[ref93] Tellinghuisen D. J. Nowak E. J. (2003). The inability to ignore auditory distractors as a function of visual task perceptual load. Percept. Psychophys. 65, 817–828. doi: 10.3758/bf03194817, 12956588

[ref94] Todorov E. Jordan M. I. (2002). Optimal feedback control as a theory of motor coordination. Nat. Neurosci. 5, 1226–1235. doi: 10.1038/nn963, 12404008

[ref95] Tsal Y. Benoni H. (2010). Diluting the burden of load: perceptual load effects are simply dilution effects. J. Exp. Psychol. Hum. Percept. Perform. 36, 1645–1656. doi: 10.1037/a0018172, 20822300

[ref96] Ulrich R. Miller J. Schroter H. (2007). Testing the race model inequality: an algorithm and computer programs. Behav. Res. Methods 39, 291–302. doi: 10.3758/bf03193160, 17695357

[ref97] Van der Burg E. Olivers C. N. Bronkhorst A. W. Theeuwes J. (2008). Pip and pop: nonspatial auditory signals improve spatial visual search. J. Exp. Psychol. Hum. Percept. Perform. 34, 1053–1065. doi: 10.1037/0096-1523.34.5.1053, 18823194

[ref98] Zuanazzi A. Noppeney U. (2019). Distinct neural mechanisms of spatial attention and expectation guide perceptual inference in a multisensory world. J. Neurosci. 39, 2301–2312. doi: 10.1523/JNEUROSCI.2873-18.2019, 30659086 PMC6433765

[ref99] Zuanazzi A. Noppeney U. (2020). The intricate interplay of spatial attention and expectation: a multisensory perspective. Multisens. Res. 33, 383–416. doi: 10.1163/22134808-20201482, 31940592

