# Supplementary material for: Faster to react, slower to move: differential effects of multisensory facilitation on movement initiation and execution across the adult lifespan
Source: Front Psychol. 2026 Sep 9;17:1811531. doi: 10.3389/fpsyg.2026.1811531 (PMC13597754; doi:10.3389/fpsyg.2026.1811531)
Supplement: Supplementary file 1 [file Data_Sheet_1.PDF]

Supplementary Information

Faster to React, Slower to Move: Differential Effects of Multisensory Facilitation on Movement Initiation and Execution Across the Adult Lifespan

Ayla Barutchu[1,2] & Charles Spence[1]

[1]Department of Experimental Psychology, University of Oxford, Oxford, United Kingdom

[2]Institute of Mental and Physical Health and Clinical Translation (IMPACT), Deakin University, Australia

Supplementary Information A

Tap Distance for the Visual Discrimination Task

In the visual discrimination task, participants accurately tapped within the boundaries of the visual target objects, which measured 25 mm^2. Only accurate trials were included in this analysis, and tap RTs that were more than 3 SD from each participant's average tap location were defined as error trials. On average, the tap distance from the center of the target was less than 10 mm (see Figure A1). A 2(target side: left, right) × 2(distractor: present, absent) × 3(auditory stimulus: none, auditory congruent, auditory incongruent) × 3(age group: <30yo, 50–69yo, and ≥70yo) mixed ANOVA revealed a significant main effect of distractor only, $F(1, 46) = 21.80$, $p < .001$, partial $\eta^2 = .32$. Follow-up main effects analyses showed that participants tapped significantly closer to the center of the targets in the absence of distractors, although the difference was small (< 2 mm) and well within the object boundaries, indicating very high spatial precision across all age groups despite the brief 200 ms presentation of the targets. No other main effects or interaction effects approached significance ($p > .1$ for all).

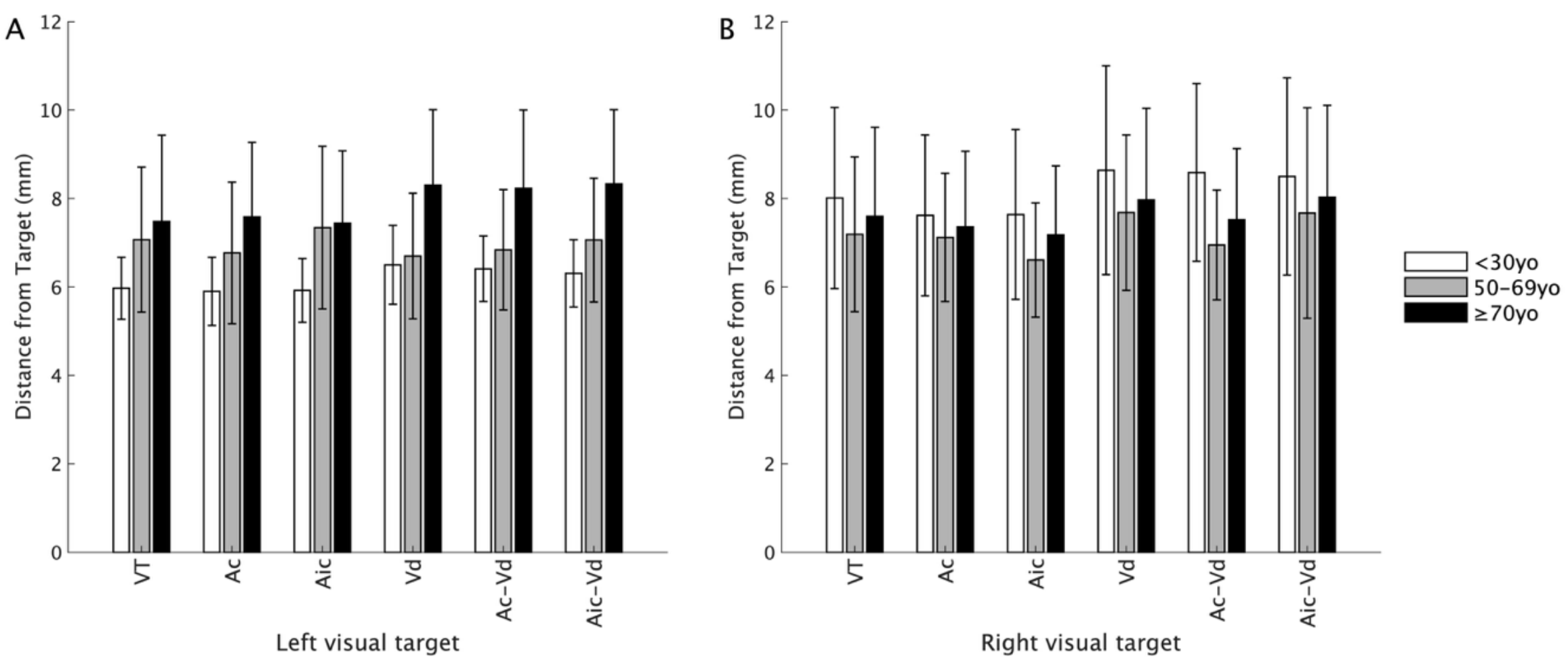

Figure A1. Means (*M*) for tap distances from the central point of the visual target (mm) presented to the left (**A**) and right (**B**) visual field for the three groups (<30yo, 50–69yo, and ≥70yo) and stimulus condition: Visual Target (VT) with spatially congruent (Ac) and incongruent auditory (Aic) stimuli and visual distractor (Vd). Error bars represent 95% *CI*.

Supplementary Information B

Analysis of Tap Responses for the Detection Task

Consistent with the lift RTs presented in the main manuscript, the fastest tap times were observed for multisensory stimuli (see Figure B1).

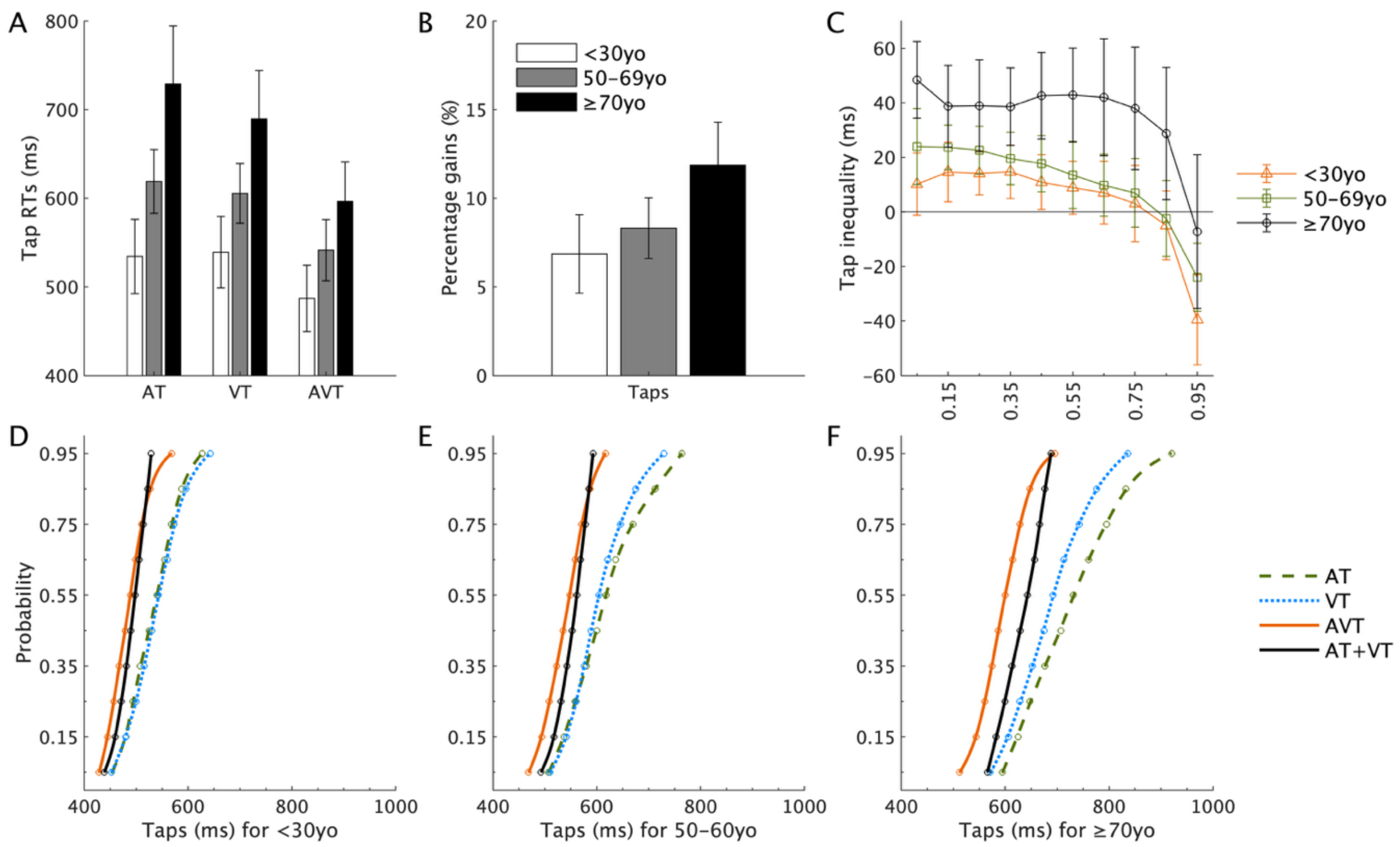

Figure B1. Tap responses on the detection task. **A.** Mean lift RTs (ms) and **B.** mean percentage gains for auditory (AT), visual (VT) and audiovisual (AVT) stimuli for each group: <30yo (white), 50-69yo (grey) and ≥70yo (black). Percentage (%) multisensory gains were calculated relative to the faster of the unisensory conditions. **C.** Inequality in ms between AVT CDF and AT+VT CDF with positive values representing race-model violations. Cumulative density functions (CDFs) for tap RTs for AT, VT, AVT and AT+VT CDFs for <30yo (**D**), 50-69yo (**E**) and ≥70yo (**F**). Error bars represent 95% *CI*.

A 3 (age group: <30yo, 50–69yo, and ≥70yo) × 3 (stimulus type: AT, VT, and AVT) mixed-design ANOVA was conducted on tap RTs. Consistent with the lift RT results reported in the main text, there were significant main effects of stimulus type, $F(1.52, 77.46) = 71.46$,

$p < .001$, partial $\eta^2 = .58$, and age group, $F(2, 51) = 14.06$, $p < .001$, partial $\eta^2 = .35$. The Age Group × Stimulus Type interaction was also significant, $F(3.04, 77.46) = 5.52$, $p = .002$, partial $\eta^2 = .18$. Follow-up analyses showed that the <30yo group responded significantly faster than both older groups for the unisensory AT and VT conditions (all *p*s < .02). For the multisensory AVT condition, only the <30yo and ≥70yo groups differed significantly ($p < .001$). Within each age group, AVT produced the fastest tap RTs. In the <30yo and 50–69yo groups, AVT was significantly faster than both AT and VT (all $p < .001$), whereas AT and VT did not differ. In the ≥70yo group, all three stimulus types differed significantly, with AVT producing the fastest responses (both $p < .001$) and VT also being significantly faster than AT ($p = .02$). These findings closely mirrored the lift RT analyses presented in the main manuscript.

Percentage multisensory gains for tap RTs also differed significantly across age groups, as shown by a one-way factorial ANOVA, $F(2, 51) = 6.35$, $p = .003$, partial $\eta^2 = .20$. Follow-up comparisons showed that the ≥70yo group exhibited significantly greater percentage gains than both the <30yo ($p = .004$) and 50–69yo ($p = .045$) groups, whereas the two younger groups did not differ significantly ($p = .72$).

Consistent with the lift RT analyses, race model violations were observed for tap RTs below the 0.55 cumulative probability for all three age groups (all $p < .001$; Figure B1). A 3 (age group: <30yo, 50–69yo, and ≥70yo) × 5 (probability) mixed-design ANOVA revealed a significant main effect of age group for tap RTs, $F(2, 50) = 9.86$, $p < .001$, partial $\eta^2 = .28$. Follow-up analyses showed that race model violations were significantly greater in the ≥70yo group than in both the <30yo and 50–69yo groups across the fastest portion of the tap RT distribution (all $p < .05$).

Supplementary Information C

The Effect of Switch Costs in the Detection Task

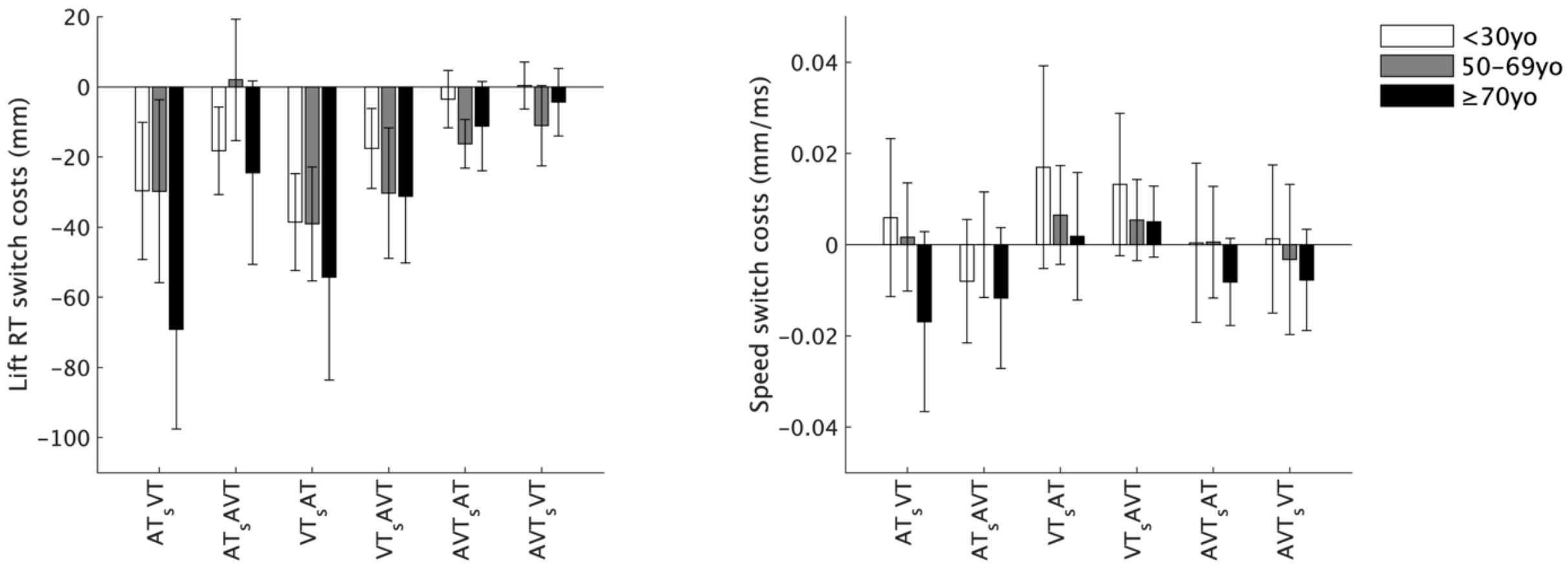

Figure C1. Switch Costs in the Detection Task. Auditory (AT), visual (VT) and Audiovisual (AVT) target switch (s) costs (±*CI*) for Lift RTs and speed for each age group: <30yo, 50–69yo, and ≥70yo. Here, 's' denotes the previous stimulus: AT sVT indicates the switch cost for AT targets when the preceding trial was VT.

The enhanced multisensory facilitation observed in older adults may be partly driven by sensory switching effects (e.g., Barutchu & Spence, 2020; Barutchu & Spence, 2021; Kreutzfeldt et al., 2015; Otto & Mamassian, 2012). Switch costs were not a primary focus of the present study, as the paradigm was intentionally designed to be manageable for individuals with less stable selective attention. Trial counts were reduced following piloting to ensure that older participants could sustain attention and complete both tasks. Auditory (AT), visual (VT), and audiovisual (AVT) targets were presented with equal probability across 90 trials (30 per condition). After excluding errors and outliers, approximately six to ten trials remained per switch transition, providing insufficient data for comprehensive analyses of sensory switch costs. Nevertheless, exploratory analyses of lift RTs and movement speed were conducted. Tap timing effects closely paralleled lift RTs. Therefore, Figure C1 shows lift RTs and movement speed only. Despite the limited sample size per participant and condition, the observed effects were consistent with previous findings (Figure C1).

Lift RT switch costs were remarkably consistent across participants. Older adults (≥70 years) exhibited larger switch costs when alternating between unisensory targets (ATsVT, VTsAT) than when switching from multisensory to unisensory targets (AVTsVT, AVTsAT), replicating prior work (Barutchu & Spence, 2021). The effects of switching on movement speed did not mirror those observed for lift RTs (Figure B1). Movement speed appeared less affected by switching, particularly in young adults, suggesting that switch costs primarily influence action initiation rather than execution. Accordingly, in older adults, unisensory switching disproportionately slowed responses and amplified the apparent advantage on multisensory trials, implying that enhanced multisensory facilitation may partially buffer sensory switch costs. Here, “s” denotes the preceding stimulus; for example, ATsVT refers to the cost for an AT trial that followed a VT stimulus.

Supplementary Information D

Analysis of All Motor Responses for Left/Right Stimuli on the Discrimination Task

The fastest lift and tap responses were observed for multisensory stimuli with no visual distractor (see Figure D1). Interestingly, participants were also faster to respond to visual targets to the right than the left of the screen.

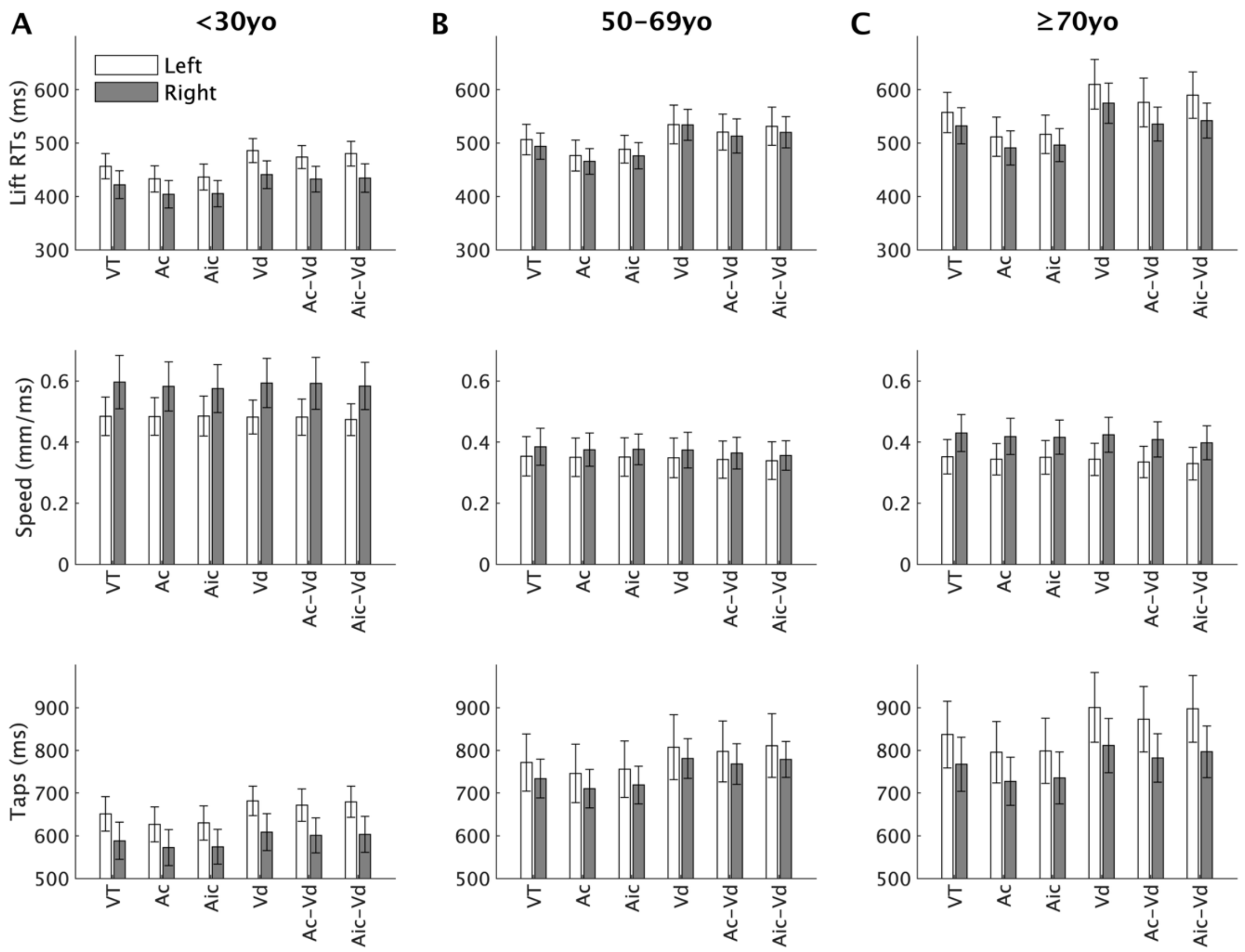

Figure D1. Mean lift RT, speed and tap times for <30yo (**A**), 50-69yo (**B**), and ≥70yo age groups (**C**) for visual targets (VT) presented with and without visual distractors (Vd), and with spatially congruent (Ac) and incongruent (Aic) auditory stimuli for each age group: <30yo, 50–69yo, and ≥70yo. Error bars represent 95% *CI*.

Table D1

Significance for main and interaction effects, degrees of freedom (DF) and partial η^2 for lift RTs, speed and tap RTs analyzed using a 2(Target Side: left and right) x 2(Distractor: present and absent) x 3(Auditory stimulus: Non, Ac and Aic) x 3(age groups: <30yo, 50-69yo, and ≥70yo) mixed ANOVA.

	Lift RTs				Speed				Tap RTs			
	DF	*F*	*p*	η^2	*DF*	*F*	*p*	η^2	*DF*	*F*	*p*	η^2
Group	**2,46**	**14.09**	**<.001***	**.38**	**2,46**	**11.89**	**<.001***	**.34**	**2,46**	**13.80**	**<.001***	**.38**
Aud	**1.4,65.2**	**89.84**	**<.001***	**.66**	**1.5,69.8**	**24.70**	**<.001***	**.35**	**1.8,80.8**	**56.51**	**<.001***	**.55**
Dis	**1,46**	**147.54**	**<.001***	**.76**	**1,46**	**8.67**	**.005***	**.16**	**1,46**	**143.02**	**<.001***	**.76**
Side	**1,46**	**61.23**	**<.001***	**.57**	**1,46**	**42.33**	**<.001***	**.48**	**1,46**	**59.56**	**<.001***	**.56**
Group*Aud	**4,92**	**8.47**	**<.001***	**.27**	4,92	0.91	.46	.09	**4,92**	**5.11**	**<.001***	**.18**
Group*Dis	**2,46**	**3.56**	**.04***	**.13**	2,46	2.13	.13	.09	**2,46**	**5.22**	**.009***	**.19**
Group*Side	**2,46**	**6.48**	**.003***	**.22**	**2,46**	**4.94**	**.01***	**.18**	2,46	3.21	.05	.12
Aud*Side	2,92	0.56	.57	.01	**2,92**	**6.28**	**.003***	**.12**	2,92	.33	.72	.01
Aud*Dis	**2,92**	**9.45**	**<.001***	**.17**	**2,92**	**4.18**	**.02***	**.08**	**2,92**	**23.16**	**<.001***	**.34**
Dis*Side	**1,46**	**6.81**	**.01***	**.13**	1,46	0.66	.42	.01	**1,46**	**6.80**	**.01***	**.13**
Group*Aud*Dis	4,92	0.32	.87	.01	4,92	1.15	.34	<.05	4,92	1.27	.29	.05
Group*Aud*Side	4,92	0.31	.87	.01	4,92	0.19	.94	.01	4,92	0.18	.94	.01
Group*Side*Dis	**2,46**	**4.80**	**.01***	**.17**	2,46	2.81	.07	.11	2,46	4.85	.091	.17
Aud*Dis*Side	2,92	2.09	.13	.04	2,92	0.75	.48	.02	2,92	2.05	.13	.04
Group*Aud*Dis*Side	4,92	0.26	.90	.01	4,92	1.28	.28	.05	4,92	0.30	.88	.01

Note. Dis = Distractor; Aud = Auditory stimulus. *Significant results for main and interaction effects are depicted with * and appear in bold.*

Lift RTs, Tap RT and speed were initially analyzed using a 2(target side: left and right) x 2(distractor: present and absent) x 3(Auditory stimulus: Non, Ac and Aic) x 3(age groups: <30yo, 50-69yo, and ≥70yo) mixed ANOVA, which showed robust main effects for age group, target side, distractors, and auditory stimulation for lift RTs and Speed (see Table 1). Follow-up analyses of the significant interactions showed that distractors significantly slowed both lift RTs and tap RTs for both target sides and in all age groups (all $p < .001$). Responses were significantly faster to right- than left-sided targets, both with and without distractors (all $p < .02$), except in the 50–69yo group when distractors were present. In addition, for left target presentations only the <30yo and ≥70yo groups differed significantly ($p < .001$), whereas for right target presentations the <30yo group responded significantly faster than both older groups (both $p < .01$). Audiovisual facilitation was evident for both lift RTs and tap RTs across all age groups, with responses significantly faster for Ac than Aic and significantly faster for Aic than VT (all $p \leq .009$). Across all auditory conditions,

distractor-related slowing increased with age, with the largest distractor costs observed in the ≥70yo group and the smallest in the <30yo group. Overall, tap RTs closely paralleled the lift RT findings, although age-related lateralisation effects were less pronounced and the influence of auditory congruency under distractor conditions was relatively greater by movement completion.

Movement speed showed a different pattern. Follow-up analyses of the significant Auditory Stimulus × Target Side interaction showed that movement speed was significantly faster for right- than left-sided targets across all auditory conditions (all $p < .001$). Movement speed for right-sided targets also slowed more in the presence of auditory stimuli, particularly when the auditory stimulus was spatially incongruent. Follow-up analyses of the significant Distractor × Auditory Stimulus interaction showed that distractors significantly slowed movement speed in the congruent ($p = .030$) and incongruent ($p = .001$) auditory conditions, but not in the visual-only condition ($p = .074$). In the absence of distractors, both audiovisual conditions slowed movement speed relative to the visual-only condition but did not differ from each other. When distractors were present, all three auditory conditions differed significantly, with movement speed slowing progressively from the visual-only (VT) to the congruent (Ac) and incongruent (Aic) auditory conditions.

The faster motor responses to right-sided targets likely reflect participants' handedness (see Figure D2). The overall pattern of right-side bias was relatively consistent across both the auditory and distractor conditions (Figure D1). Therefore, responses to targets presented on the right and left hemifields were pooled for the main analyses presented in the manuscript and subsequent analyses of multisensory gains and distractor costs.

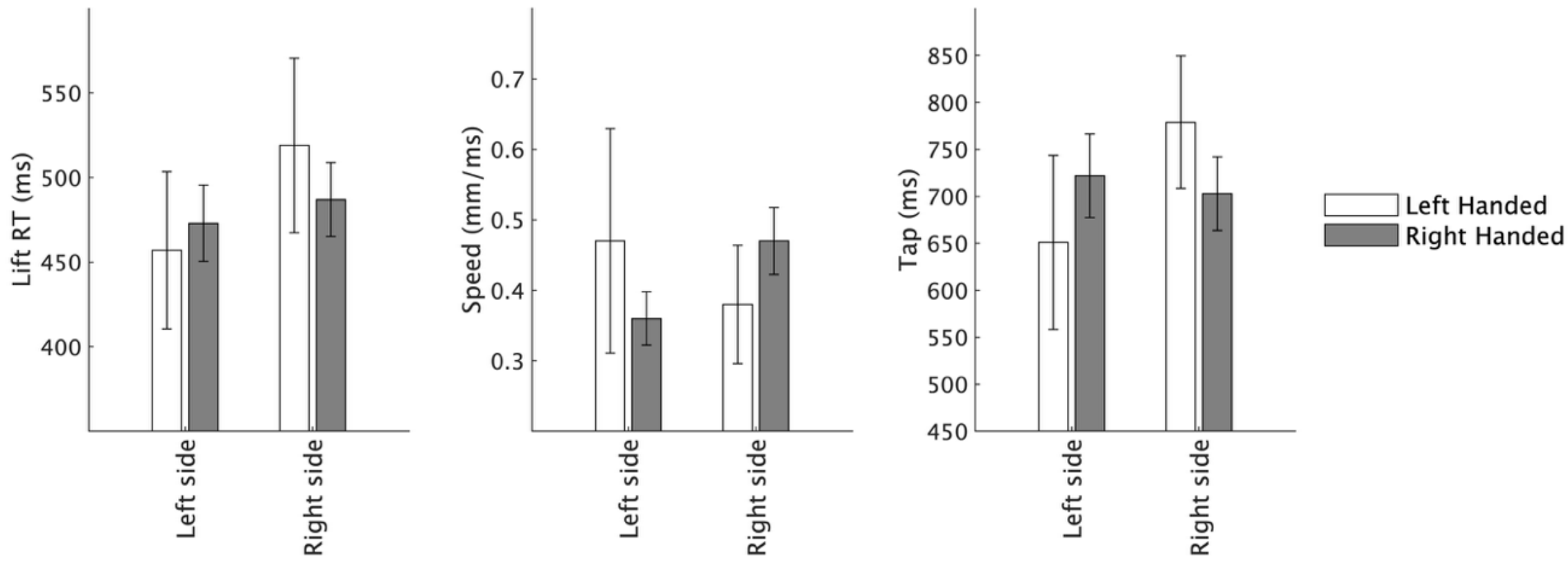

Figure D2. Lift RTs, speed and tap RTs (±*CI*) for left- and right-side targets and left-handed and right-handed participants.

We explored whether the observed right-side motor bias was related to handedness by collapsing responses across conditions and averaging left- versus right-side targets. In our sample, five participants were left-handed; for these participants, lift RTs, tap times, and movement speed were faster for targets on the left side of the screen (Figure C1). Among the remaining right-handed participants (N = 44), the reverse pattern was evident across all three measures. Given the small number of left-handed participants, we did not conduct subgroup inferential tests. Nevertheless, this pattern aligns with prior reports (e.g., Daini et al., 2018). The influence of handedness on spatially lateralized multisensory effects warrants further investigation in larger, pre-stratified samples.

Supplementary Information E

Visual Discrimination Task: Multisensory Gains and Costs from Baseline

For the visual discrimination task, we used one-sample *t*-tests against zero to assess for significant gains and costs within each group (Table E1). Šidák corrections were applied separately for each age group and measure (lift RTs, speed and tap RTs). Significant multisensory gains in lift reaction times and tap RTs were observed across all age groups for both the auditory congruent and incongruent conditions (Ac and Aic). Distractor-related costs emerged for the visual distractor condition (Vd) and for the auditory incongruent condition combined with the visual distractor (Aic-Vd), but not for the congruent auditory stimulus paired with the visual distractor (Ac-Vd), except in the 50–69yo group. This pattern of results suggests that congruent auditory signals provided some protection against the disruptive effects of visual distractors on lift reaction times and tap RTs. For movement speed, no significant distractor costs were found in the <30yo group. In the 50–69yo group, however, significant slowing was observed across all three distractor conditions (Vd, Ac-Vd, and Aic-Vd). The ≥70yo group showed significant slowing for both audiovisual distractor conditions (Aic-Vd and Ac-Vd).

Table E1

*T-values and p-values of one-sample t-tests against zero for gains and costs for auditory and distractor conditions, respectively, in the visual discrimination task within each age group. * = significant t-test after Šidák corrections for multiple comparisons.*

		Lift RTs		Speed		Tap RTs	
		t	*p*	*t*	*p*	*t*	*p*
<30yo	Vd	**-6.43**	**<.001***	0.06	.48	**-5.30**	**<.001***
DF = 16	Ac	**5.21**	**<.001***	-1.17	.26	**4.09**	**<.001***
	Aic	**7.87**	**<.001***	-1.62	.13	**5.68**	**<.001***
	Ac-Vd	**3.49**	**.002***	-0.83	.21	2.77	.007*
	Aic-Vd	**2.97**	**.005***	-2.86	.006	1.83	.04
50–69yo	Vd	**-6.66**	**<.001***	-3.45	.002	**-7.20**	**<.001***
DF = 16	Ac	**6.49**	**<.001***	-1.82	.09	**5.13**	**<.001***
	Aic	**3.26**	**<.004***	-1.07	.30	**5.13**	**<.005***
	Ac-Vd	**3.65**	**.001***	-2.32	.01	2.22	.02
	Aic-Vd	1.52	.07	**-2.66**	**.009***	-0.42	.34
≥*70yo*	Vd	**-6.63**	**<.001***	0.90	.19	**-5.71**	**<.001***
DF = 17	Ac	**7.04**	**<.001***	**-3.10**	**.007***	**5.92**	**<.001***
	Aic	**11.04**	**<.001***	-1.99	.06	**6.92**	**<.001***
	Ac-Vd	**7.42**	**<.001***	**-4.18**	**<.001***	-8.54	**<.001***
	Aic-Vd	**4.94**	**<.001***	**-5.01**	**<.001***	-2.03	.06

Note. Ac = Auditory congruent, Aic = Auditory incongruent, Vd = visual distractor

Supplementary Information F

Visual Discrimination Task: Multisensory Gains for Tap RTs

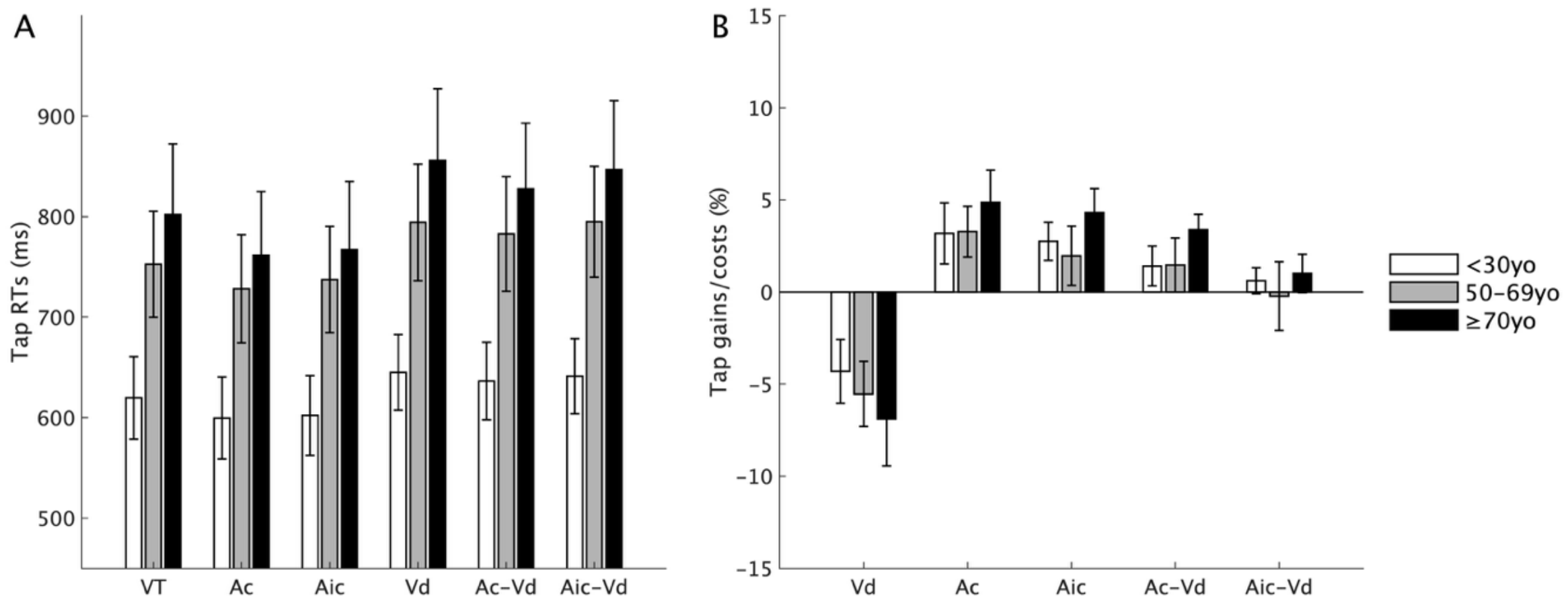

Figure F1. **A**. Mean tap responses (left and right filed combined) on the visual discrimination task for visual targets (VT) presented with and without visual distractors (Vd), and with spatially congruent (Ac) and incongruent (Aic) auditory stimuli for each age group: <30yo, 50–69yo, and ≥70yo. **B**. Percentage gains and costs for tap RTs for the same conditions. Positive values represent multisensory gains whereas negative values represent costs, calculated relative to the corresponding unisensory condition (i.e., Ac and Aic relative to VT, and Ac-Vd and Aic-Vd relative to Vd). For the Vd condition, negative values represent the cost of the visual distractor relative to VT. Error bars represent 95% confidence intervals (CIs).

Consistent with the lift RT findings, the fastest tap RTs were observed for multisensory stimuli presented without a visual distractor (Figures F1 and F2). Tap RTs were analysed using a 2 (distractor: present, absent) × 3 (auditory stimulus: VT, Ac, and Aic) × 3 (age group: <30yo, 50–69yo, and ≥70yo) mixed-design ANOVA. There were significant main effects of age group, $F(2, 46) = 13.80$, $p < .001$, partial $\eta^2 = .38$, distractor, $F(1, 46) = 143.02$, $p < .001$, partial $\eta^2 = .76$, and auditory stimulus, $F(1.76, 80.82) = 56.15$, $p < .001$, partial $\eta^2 = .55$. Follow-up analyses showed that the <30yo group responded significantly faster than both the 50–69yo and ≥70yo groups, whereas the two older groups did not differ significantly. Distractors significantly slowed tap RTs ($p < .001$), and all three auditory conditions differed significantly from one another (all $p < .001$), with the fastest responses

observed for the spatially congruent audiovisual condition (Ac), followed by the spatially incongruent audiovisual condition (Aic), and the slowest responses for the visual-only (VT) condition. There were also significant Age Group × Distractor, $F(2, 46) = 5.22$, $p = .009$, partial $\eta^2 = .19$, Age Group × Auditory Stimulus, $F(3.51, 80.82) = 5.11$, $p = .002$, partial $\eta^2 = .18$, and Distractor × Auditory Stimulus interactions, $F(2, 92) = 23.16$, $p < .001$, partial $\eta^2 = .34$, whereas the three-way interaction was not significant, $F(4, 92) = 1.27$, $p = .29$, partial $\eta^2 = .05$. Follow-up simple effects analyses showed that distractors significantly slowed tap RTs in all three age groups (all $p < .001$). The significant interactions reflected differences in the magnitude, rather than the overall pattern, of these effects. Specifically, in the absence of distractors, responses were significantly faster for Ac than Aic and significantly faster for Aic than VT (all $p < .001$), whereas in the presence of distractors, Ac remained significantly faster than both VT and Aic (all $p < .001$), while VT and Aic no longer differed significantly. Across all auditory conditions, distractor-related slowing increased with age, with the largest distractor costs observed in the ≥70yo group and the smallest in the <30yo group.

A 5 (stimulus type: Vd, Ac, Aic, Ac-Vd and Aic-Vd) × 3 (age group: <30yo, 50–69yo, and ≥70yo) mixed-design ANOVA was conducted to examine multisensory and distractor-related gains and costs in tap RTs (Figure F1B). There was a significant main effect of stimulus type, $F(2.42, 111.16) = 117.10$, $p < .001$, partial $\eta^2 = .72$, and a significant Age Group × Stimulus Type interaction, $F(4.83, 111.16) = 2.93$, $p = .02$, partial $\eta^2 = .11$, whereas the main effect of age group was not significant, $F(2, 46) = 1.43$, $p = .25$, partial $\eta^2 = .06$. Follow-up analyses indicated that age-related differences were confined to the distractor-related conditions. Specifically, the ≥70yo group exhibited significantly greater gains than the 50–69yo group for the Vd ($p = .03$), Ac-Vd ($p = .03$), and Aic-Vd ($p = .04$) conditions, whereas no other age-group differences were observed. Across all three age groups, both the Ac and Aic conditions produced significantly greater gains than the Vd condition (all $p < .001$) and did not differ significantly from each other. The influence of audiovisual spatial congruency was confined to the distractor conditions. Specifically, the Aic-Vd condition produced significantly smaller gains than the Ac condition in all three age groups (all $p \leq$

.03), whereas the Ac-Vd and Aic-Vd conditions differed significantly only in the 50–69yo (p = .02) and ≥70yo ($p < .001$) groups. Overall, the pattern of percentage gains for tap RTs was broadly similar to that observed for lift RTs reported in the main manuscript.

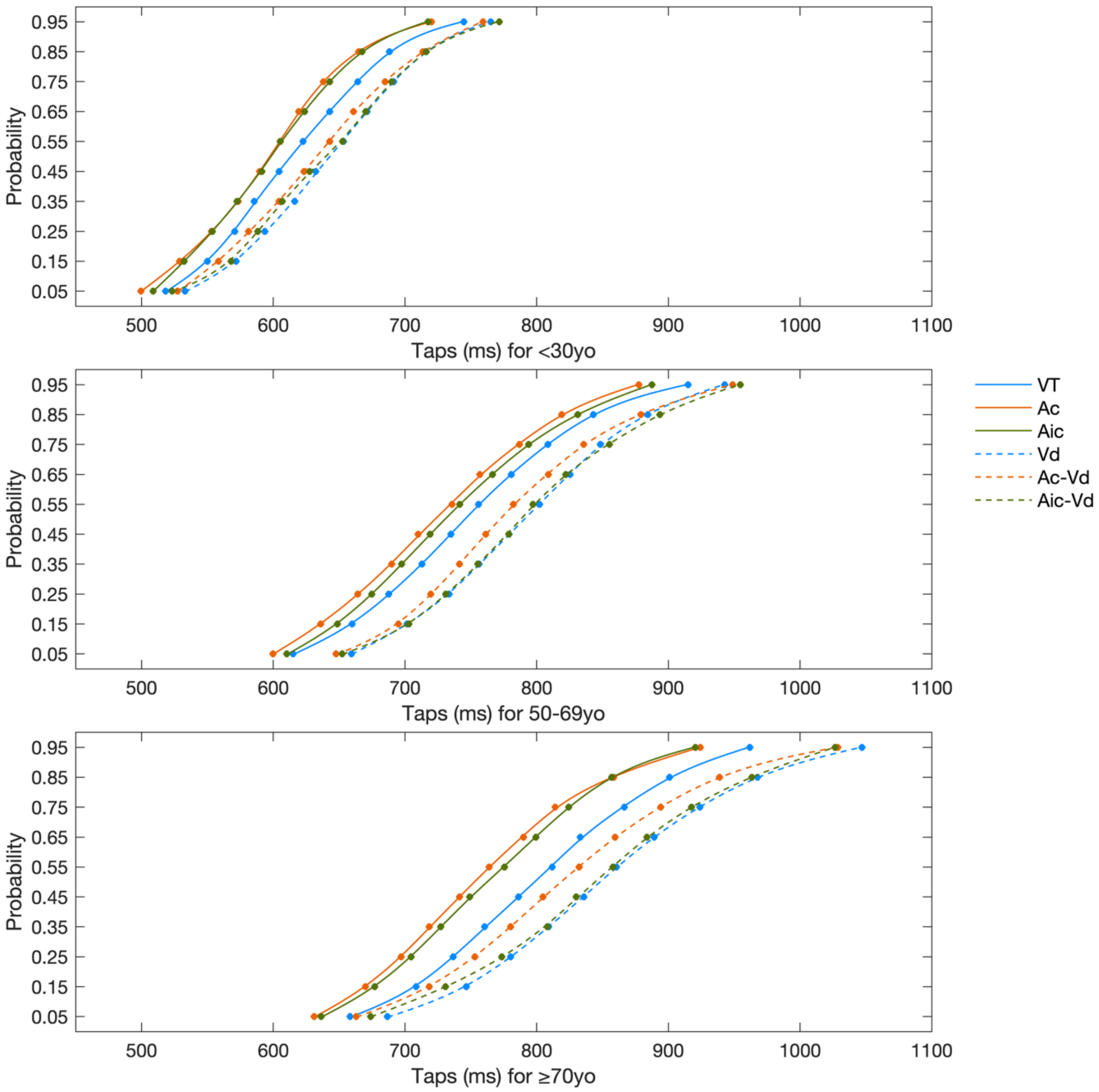

Figure F2. Cumulative density functions (CDFs) for tap RTs to visual targets in the discrimination task, collapsed across left- and right-sided target presentations. CDFs are shown for visual targets presented alone (VT) or combined with spatially congruent (Ac) or spatially incongruent (Aic) auditory stimuli, with and without a visual distractor (Vd), for each age group.

References

Barutchu, A., & Spence, C. (2020). An experimenter's influence on motor enhancements: The effects of letter congruency and sensory switch-costs on multisensory integration. *Frontiers in Psychology*, *11*, 588343. https://doi.org/10.3389/fpsyg.2020.588343

Barutchu, A., & Spence, C. (2021). Top-down task-specific determinants of multisensory motor reaction time enhancements and sensory switch costs. *Experimental Brain Research*, *239*(3), 1021–1034. https://doi.org/10.1007/s00221-020-06014-3

Daini, R., Vallar, G., & Arduino, L. S. (2018). Why we move to the right? The dominant hand motor-spatial bias. *Journal of Experimental Psychology: General*, *147*(10), 1488–1502. https://doi.org/10.1037/xge0000476

Kreutzfeldt, M., Stephan, D. N., Sturm, W., Willmes, K., & Koch, I. (2015). The role of crossmodal competition and dimensional overlap in crossmodal attention switching. *Acta Psychologica*, *155*, 67–76. https://doi.org/10.1016/j.actpsy.2014.12.006

Otto, T. U., & Mamassian, P. (2012). Noise and correlations in parallel perceptual decision making. *Current Biology*, *22*(15), 1391–1396. https://doi.org/10.1016/j.cub.2012.05.031
